# Characterization of the caleosin gene family in the Triticeae

**DOI:** 10.1186/1471-2164-15-239

**Published:** 2014-03-27

**Authors:** Hala Badr Khalil, Sabrina C Brunetti, Uyen Minh Pham, Deborah Maret, André Laroche, Patrick J Gulick

**Affiliations:** Biology Department, Concordia University, 7141 Sherbrooke W, Montreal, QC H4B 1R6 Canada; Agriculture and Agri-Food Canada, Lethbridge Research Centre, 5403, 1st Avenue South, C.P. 3000, Lethbridge, AB T1J 4B1 Canada; Department of Genetics, Faculty of Agriculture, Ain-Shams University, Shoubra El-khema, Cairo, Egypt

**Keywords:** Caleosin gene family, Calcium-binding protein, Phylogenetic analysis, Tissue-specific expression, GAP, Gα, Heterotrimeric G protein signaling, RNA-seq

## Abstract

**Background:**

The caleosin genes encode proteins with a single conserved EF hand calcium-binding domain and comprise small gene families found in a wide range of plant species. Some members of the gene family have been shown to be upregulated by environmental stresses including low water availability and high salinity. Caleosin 3 from wheat has been shown to interact with the α-subunit of the heterotrimeric G proteins, and to act as a GTPase activating protein (GAP). This study characterizes the size and diversity of the gene family in wheat and related species and characterizes the differential tissue-specific expression of members of the gene family.

**Results:**

A total of 34 gene family members that belong to eleven paralogous groups of caleosins were identified in the hexaploid bread wheat, *T. aestivum.* Each group was represented by three homeologous copies of the gene located on corresponding homeologous chromosomes, except the caleosin 10, which has four gene copies. Ten gene family members were identified in diploid barley, *Hordeum vulgare*, and in rye, *Secale cereale,* seven in *Brachypodium distachyon*, and six in rice, *Oryza sativa*. The analysis of gene expression was assayed in triticale and rye by RNA-Seq analysis of 454 sequence sets and members of the gene family were found to have diverse patterns of gene expression in the different tissues that were sampled in rye and in triticale, the hybrid hexaploid species derived from wheat and rye. Expression of the gene family in wheat and barley was also previously determined by microarray analysis, and changes in expression during development and in response to environmental stresses are presented.

**Conclusions:**

The caleosin gene family had a greater degree of expansion in the Triticeae than in the other monocot species, *Brachypodium* and rice. The prior implication of one member of the gene family in the stress response and heterotrimeric G protein signaling, points to the potential importance of the caleosin gene family. The complexity of the family and differential expression in various tissues and under conditions of abiotic stress suggests the possibility that caleosin family members may play diverse roles in signaling and development that warrants further investigation.

**Electronic supplementary material:**

The online version of this article (doi:10.1186/1471-2164-15-239) contains supplementary material, which is available to authorized users.

## Background

Caleosins are calcium-binding proteins encoded by small gene families in plants, and some members of the gene family have been shown to play an important role in signaling and in the response to stress. Ta-*Clo3* encoded by a member of this gene family in wheat (*Triticum aestivum),* was shown to have GAP activity with the heterotrimeric G protein subunit, Gα [1]. The caleosins do not have significant sequence similarity with the Regulators of G protein Signaling (RGS) in other species and appear to be a new class of proteins that act as heterotrimeric G protein GAPs. The wheat Clo3 was also shown to interact with phosphoinositide-specific phospholipase C1 (PI-PLC1) *in vivo* and *in vitro*, and the interaction between Clo3, Gα and PI-PLC1 was found to be competitive [1]. A homolog of Ta-*Clo3* in Arabidopsis, At-*Clo3*, also known as Responsive to Dehydration 20 (RD20), has been shown to be strongly induced by drought, abscisic acid and high salinity and was experimentally shown to bind calcium [2]. The *clo3*/*rd20* mutants in Arabidopsis showed enhanced sensitivity to drought conditions and *RD20* was implicated in the control of stomata aperture, reduction of growth, and increased transpirational efficiency [3]. Plants’ responses to water deficit are known to activate signal transduction cascades that increase the level of secondary messengers, including calcium, thus some members of the caleosin gene family appear to play a critical role in water deficit signaling and to link calcium regulation to G protein signaling. Analysis of caleosins in barley also suggests a role in lipid trafficking and membrane expansion [4]. The caleosin assembled oil bodies have been proposed as useful components of a nano-carrier for therapeutic purposes, and have been specifically used as drug carriers, targeting cancer cells [5]. It is unknown if the role of caleosins in the stress response is related to their role in lipid bodies, or if they are simply different functions carried out by different members of the gene families. Caleosins comprise a gene family of seven members in Arabidopsis and the rice genome contains five gene family members.

Bread wheat, *Triticum aestivum*, is one of the most important cereal species grown world-wide. It is an allohexaploid, derived from two polyploidization events. The first hybridization between the diploid *T. urartu* and an unknown species thought to be closely related to *Aegilops speltoides*, which contributed the A and B genomes, respectively, occurred approximately 500,000 years ago. The tetraploid species was domesticated as *T. turgidum*, commonly known as pasta wheat. The second polyploidization occurred between *T. turgidum* and *Aegilops tauchii*, the D genome progenitor, approximately 8,000 years ago. *Hordeum vulgare*, barley, and *Secale cereale*, rye, are closely related crop species that belong to the tribe Triticeae, estimated to have diverged from the *Triticum-Aegilops* lineage 11 and 7 MYA, respectively [6]. Hexaploid triticale (x *Triticosecale*) is a synthetic hybrid crop species first developed in the late 19^th^ century by crosses between *Secale cereale* and *T. turgidum*, and contains A, B, and R genomes [7]. Triticale, grown largely for livestock feed, as well as for human consumption, has an important potential as a crop, especially under conditions that are less favorable for wheat cultivation, such as marginal soils in drought-prone regions. Generally, it combines the grain quality and yield potential of wheat with the environmental stress and disease tolerance of rye [7]. Compared to wheat, triticale appears to have a higher resistance to many wheat fungal diseases and pests, as well as viral diseases. In addition to tolerance to conditions of drought, triticale varieties are able to adapt to stress conditions such as excess moisture and acidic soils [7]. Triticale is also an important model for investigation of the rapid changes involving genomic remodeling and changes in gene expression subsequent to polyploidization.

In order to facilitate the analysis of the members of the caleosin gene family and investigate the diverse roles these proteins may play in signaling, we report a description of the whole gene family in hexaploid wheat, diploid rye, and triticale, based on analysis of high-throughput cDNA sequencing data sets and compare these to the other diploid species including barley (*Hordeum vulgare)*, *Brachypodium distachyon*, rice, and Arabidopsis.

## Methods

### Caleosin contigs assembly

#### Triticum aestivum

The calcium binding protein Ta-Clo3/J900 from wheat was used for BLASTp [8] searches in the GenBank NR databases for related caleosin gene sequences in Arabidopsis and rice. The complete set of Arabidopsis and rice caleosin amino acid sequences, as well as that of the Ta-Clo3 were then used to search, with tBLASTn, for related sequences in the GenBank EST database for *T. aestivum*. The EST sequences were assembled into contigs using CAP3 [9] under high stringency parameters of a minimum sequence identity of 98%; minimum overlap length of 40 nt; gap penalty, 6; match value, 2; mismatch penalty (−5). Open reading frames and translation of the contigs were carried out with the ExPASy translate tool [10] and confirmed by BLASTx with the GenBank NR database comparison to related sequences, as well as by comparison among the *T. aestivum* caleosin sequences as the data set was developed. Contigs were manually edited to obtain full length cDNA sequences by first identifying any partial length contigs by BLASTx to the NR database and then identify additional ESTs with 100% identity and a minimum overlap of 120 nt in the *T. aestivum* EST database at NCBI. ESTs were selected which could extend the 5′ or 3′ end of the initial contig. CAP3 was used to assemble all ESTs together with partial sequences to generate full-length contigs. After an initial set of contigs were completed, the process was reiterated to identify additional ESTs and additional homeologous members of the gene family that were not represented in the initial contig set. The *T. aestivum* caleosin contigs were used to search the Wheat Survey Sequences (WSS) of the International Wheat Genome Sequencing Consortium [11] in order to identify the chromosome or chromosome arm on which the gene was located. One cDNA, *Clo4-B*, not represented in the *T. aestivum* GenBank EST database, was assembled independently from 454 (Roche) cDNA sequences of triticale and from matches of the homeologous *Clo4-A* to the WSS genomic wheat sequence database.

#### Secale cereale

Eleven paralogous wheat caleosin genes were used to search for orthologs in five 454-cDNA rye libraries obtained from anther, pistil, crown, roots, and stem, using BLASTn. All 454-cDNAs that matched to wheat caleosin genes with a minimum overlap of 100 nt were selected as caleosin homologs regardless of their BLASTn scores or percent identities. The selected candidates of rye 454-cDNAs were assembled using CAP3 with the same parameters as described above for *T. aestivum*, except the minimum overlap length was set at 35 nt. Using these high stringency assembly parameters, the CAP3 assembly yielded 45 contigs. The preliminary set of contigs was searched with the eleven wheat paralogs using BLASTn to eliminate duplicates and to select contigs with the longest contig length and highest sequence similarity. Contigs assembled with a low depth of coverage were individually compared to the rye 454-cDNAs to confirm the accuracy of their assemblies.

#### Hordeum vulgare

The 11 paralogous caleosin gene sequences of wheat were used to search in the GeneBank databases for homologous genes in barley (*H. vulgare)*, the best characterized member of the Triticeae. Gene sequences were identified for eight of these genes in the nr database of full length nucleotide sequences. The sequence of Hv-*Clo9* [GenBank:AK375872.1] had a frame-shift error that became apparent by comparison to the wheat orthologs; the sequences were corrected by comparison to sequences in the GenBank barley EST database. An additional barley caleosin, Hv-*Clo8*, was assembled from EST sequences in the GenBank EST database. Another contig, Hv-*Clo11* was identified in the second generation sequence database for barley [12] by a BLASTn search of the assembly 1 morex rcba database. In the latter case, the tentative barley transcript was manually assembled from the output of the BLASTn alignment of the barley subject sequence detected by the wheat Ta-*Clo11* caleosin query sequence.

#### Brachypodium distachyon

*T. aestivum* caleosin protein sequences were used to search the *B. distachyon* database [13] by tBLASTn. The complete caleosin gene sequence and coding sequences were acquired in FASTA format and were translated with the ExPASy Translate tool [10]. In cases where the original annotation appears to have misidentified the exon/intron junctions or start codons, the flanking sequence for the annotated gene region was reanalyzed and annotated manually by searching for extended ORFs and sequence similarity to the caleosin protein sequences from *T. aestivum.*

### Caleosin genes conserved domains and family phylogenetic tree

The most conserved region of the gene family was determined by using NCBI Batch Conserved Domain Search Tool [14] for all contigs, and the result was confirmed with conservation scores calculated by JasView. The calcium binding domain EF-hand motif was identified by alignment of all contigs with EF-hand motifs (1XO5, 1NYA) and calcium binding proteins (1TIZ, 1NKF, 3OX6) obtained from the Protein Data Bank [15]. The results were verified based on the EF-hand motif in Arabidopsis, described by Takahashi and coworkers [2]. The structural and functional features of caleosin genes were examined using InterPro Scan Sequence Search [16], and the Simple Modular Architecture Research Tool (SMART) [17] in genomic mode. These two programs were used in parallel to support the result from the NCBI Search Tool and to verify the presence of the EF-hand motif.

A phylogenetic tree was constructed using wheat caleosin nucleotide sequences aligned using Molecular Evolutionary Genetics Analysis (MEGA), version 5.1 [18]. The maximum likelihood method was employed based on the Jukes-Cantor model [19]. Initial tree(s) for the heuristic search were obtained automatically as follows: When the number of common sites was < 100 or less than one fourth of the total number of sites, the maximum parsimony method was used; otherwise BIONJ method with MCL distance matrix was used. A discrete Gamma distribution was used to model evolutionary rate differences among sites (5 categories (+*G*, parameter = 1.2379)). The analysis involved 35 nucleotide sequences. All positions containing gaps and missing data were eliminated. There were a total of 510 positions in the final dataset.

Multiple sequence alignment and phylogenetic tree construction for caleosins from six species were performed using MEGA, version 5.1 [18]. Sequence comparisons were based on the 174 amino acid caleosin domain of protein sequences from eleven paralogous *Clo* genes (one representative of each homeologous gene groups) of hexaploid *T. aestivum*, and the gene sequences from the diploid species: *H. vulgare*, *B. distachyon* and *S. cereale* as well as those of *O. sativa* and *A. thaliana*. The amino acid sequences from the six species were aligned using MUSCLE [20]. Phylogenetic trees were constructed using the maximum likelihood method based on the Whelan and Goldman (WAG) model [21]. This method uses standard statistical techniques for inferring probability distributions to assign probabilities to possible phylogenetic trees. The WAG model for amino acids was employed [21], an empirical model of globular protein evolution. Initial tree(s) for the heuristic search were obtained automatically as described above. A discrete Gamma distribution was used to model evolutionary rate differences among sites (5 categories (+*G*, parameter = 1.0411)). The analysis involved 54 amino acid sequences. All positions containing gaps and missing data were eliminated. There were a total of 93 amino acid positions included in the final dataset.

### Intron/exon structure of caleosin genes

The intron/exon structure of the wheat caleosin genes was determined by comparing the full length cDNA sequence to the genomic sequence in the IWGSC’s WSS database [11]. The intron/exon structure of *H. vulgare* and Brachypodium caleosin genes was similarly characterized by comparing the full length cDNA sequences to genomic sequence contigs at the Gramene database [22].

### Rye and triticale 454-cDNA library construction

#### Plant material and growth conditions

Rye (*Secale cereale)* and hexaploid triticale (*x Triticosecale* Wittm.) seedlings were grown in 15 cm diameter plastic pots containing soil-less mixture (Cornell mix) in a temperature-controlled growth chamber maintained at 20°C (day), and 18°C (night) under a photoperiod of 16 h light (250–275 μE m^−2^ s^−1^) provided by fluorescent and incandescent light. Plants were held at a constant humidity of 70% and watered daily. Specific cultivars grown, and the tissues that were harvested at specific developmental stages are listed in Additional file 1: Table S1 and Additional file 2: Table S2. When tissue was harvested it was frozen immediately in liquid nitrogen. Floral tissues from triticale and rye were harvested from plants grown as described by Tran et al. [23] at different Zadoks developmental stages [24]. For salt treatment analysis, rye cultivar Musketeer and triticale cultivar AC Certa plants were grown in hydroponic tanks containing a modified Hoagland’s solution [25] with a light cycle of 16 h light and 8 h darkness, with day/night temperatures of 22°C and 15°C, respectively. The growth solution was replaced at days seven and 14; at day 21 the hydroponic solution was replaced with fresh growth solution supplemented with 100 mM NaCl and 14 mM CaCl_2,_ treated for 24 h and harvested. For the polyethylene glycerol (PEG) treatment, three day old germinated seeds were placed on the surface of an artificial media (50 ml) containing 0%, 27%, 31% or 34% PEG 35,000 in Magenta boxes (2 seedlings/box) [26], kept in the growth chamber 30 cm beneath 40 watt Sylvania Gro-Lux Wide Spectrum lamps delivering 80 μM of light with a 16 h light period at 18°C and grown for 21 days.

#### DNA library and sequencing

Construction for the standard cDNA libraries, 0.6 mg total RNA was used to purify Poly(A)^+^ mRNA using Poly(A)Purist™ Kit (Ambion, Inc). First strand cDNA synthesis was initiated by an anchored poly (T) and SuperScript III. Then, the second strand of cDNA was made using Invitrogen reagents.

For the anther libraries [23], total RNA was extracted from rye anthers (200 mg) using the Concert™ Plant RNA Reagent (Invitrogen, Burlington, ON, Canada) according to the manufacturer’s instructions. The total RNA was further purified using the RNeasy kit (Qiagen, Mississauga, ON, Canada) following the manufacturer’s instructions. Poly A^+^ RNA was isolated from 50 μg total RNA using Dynabeads (Invitrogen, Burlington, ON, Canada) in accordance with the manufacturer’s instructions. Rye anther cDNA was generated using approximately 200 ng of poly A^+^ RNA and the SMARTer™ PCR cDNA synthesis kit (Clontech, Mountain View, CA, USA). The resulting cDNA was PCR amplified for 15 cycles. The PCR amplified cDNA was purified using the MinElute Reaction Kit (Qiagen, Mississauga, ON, Canada) and used as a template for 454 sequencing. Five micrograms of ds cDNA from the different libraries were sent to the Plant Biotechnology Institute, Saskatoon, SK, Canada, for 454 GS FLX Titanium sequencing. Root cDNA libraries from the salt stress experiments were sequenced using the same technology at Genome Quebec Innovation Centre, Montreal, PQ, Canada. The number of replicate libraries for each tissue ranged between 6 and 2 and are listed in Additional file 1: Table S1 and Additional file 2: Table S2.

### Caleosin expression analysis

The relative level of expression of caleosin gene family members in rye and in triticale was determined by analysis of Roche 454-cDNA sequence libraries. A description of the analysis is presented as a flowchart (Additional file 3: Figure S1). 454-cDNA reads were converted from 454-sff format to FASTQ format using Galaxy server from Penn State and Emory University [27]. The high quality transcripts obtained from triticale and rye tissues were aligned to their associated wheat, rye, and triticale caleosin reference sequences using the CD-HIT-EST-2D biological sequence clustering algorithm [28] using default parameters and a word size of n = 5, and a similarity cutoff of 97%. The reads that were uniquely mapped to each homeolog were selected and counted. The expression of rye and triticale cDNA reads in 454 libraries was normalized to reads per kilobase of gene length per million reads to correct the biases from differences in the gene lengths and to normalize the expression among libraries of different sizes. To characterize expression in stem tissue, cDNA libraries were made from three genotypes sampled at two times of development. Analysis by two-way ANOVA showed no significant differences in expression for caleosins among genotypes or between times of development; these six libraries were therefore used as replicates, and the data was analyzed by one-way ANOVA. The number of replicates for each of the other tissues is listed in Additional file 1: Table S1 and Additional file 2: Table S2.

The relative level of tissue-specific expression for caleosin gene family members in wheat and in barley was determined by analysis of datasets from a 61 K [29], and a 22 K [30] Affymetrix microarray, respectively, available at the PLEXdb database [31]. The relative level of gene expression in triticale in response to osmotic stress given as PEG treatments was analyzed by comparison of 454 data sets from treated and control plants. The changes in gene expression in rye roots in response to salt treatment were analyzed from 454 sequence libraries with the CD-HIT algorithm [28]. The effect of cold stress on the expression of caleosin family members was analyzed in the microarray data set of Monroy et al. [32].The effect of drought stress on caleosins were analyzed with the microarray data of Aprile et al. [33], available at the PLEXdb database [31]; the effect of ABA treatment in barley was analyzed with the microarray data set of Rodriguez et al. (GEO Accession: GSE10328), also from the PLEXdb database. The identifiers for *Clo* genes on the Affymetrix microarrays mentioned above can be found in Additional file 4.

### Statistical analysis

A two-way ANOVA was used to test for significant differences in levels of caleosin gene expression among genotypes, or between developmental stages of triticale stem and anther tissues. The statistical significance of the difference in the levels of expression among the different caleosin gene family members in each rye and triticale tissue was tested by a one-way ANOVA (p < 0.05). Duncan’s multiple range test was used to determine which caleosin genes expressed differ significantly within each tissue type. A χ2 contingency test was also used to test the significance of the difference between the level of expression of the R genome copy of caleosin genes in rye and in triticale, based on the null hypothesis that the level of individual R genome caleosin transcripts in triticale was not different than one third of the level of expression of the genes in rye. One-way ANOVA was used to test the significance of the differences in caleosin gene expression from microarray data. Duncan’s multiple range test was used to determine which caleosin genes expressed differ significantly across a panel of different tissues analyzed. Duncan’s test was also used to determine which caleosin genes expressed in barley crown tissue under control or ABA treatment conditions differ significantly. Note that the Affymetrix wheat microarray data does not distinguish between the homeologs within paralogous groups.

## Results

### Caleosin genes in *T. aestivum*

CAP3 assembly parameters ranging from 80% (default) to 99% identity were evaluated for the assembly of gene family members from hexaploid wheat and the optimal value was determined to be 98%. Assembly at a lower minimum percent identity resulted in contigs that included sequences from different homeologous gene copies. Assembly at 99% minimum identity resulted in more numerous and shorter contigs with more independent contigs for the same gene.

Among the species surveyed, hexaploid *T. aestivum* had the largest caleosin gene family with 11 gene family members per haploid genome. In total, 34 full length caleosin-like cDNA sequences were identified in this species (Table 1; Additional file 5: Figure S2). All but three of these transcript sequences were compiled from the wheat EST database at NCBI. Three additional sequences (*Clo4-B*, *Clo10-2-D*, *Clo11-A*) were obtained from the WSS derived from second generation sequences from genomic DNA. *Clo10-2-D* had a single supporting EST sequence in the GenBank EST database, and *Clo11-A* was supported by a single read from the triticale 454-cDNA data set. *Clo4-B* was identified in the WSS genomic database and the full coding region was verified by several reads from the triticale 454-cDNA data set, though *Clo4-B* from triticale has a 3 base pair deletion relative to *Clo4-B* from *T. aestivum*. Six cDNA sequences were confirmed by sequencing individual cDNA clones. Pair-wise BLASTn and ClustalW2 [34] comparison among the sequences identified 11 paralogous sets of three genes corresponding to homeologous groupings of genes derived from the three ancestral genomes of wheat (Additional file 5: Figure S2).

**Table 1:** **Caleosin genes from four species** ^**a**^

	***T. aestivum***			Homologues		
Gene	Chromosome	Nucleotide	aa	***B. distachyon*** ^***b***^	***H. vulgare*** ^***b***^	***O. sativa (Japonica)*** ^***b***^
*Clo1*	2AL	1600	302	Bradi5g15410	gi|34538472	gi297723297
*Clo1*	2BL	1154	302			
*Clo1*	2DL	1178	302			
*Clo2*	2AL	963	244	Bradi5g15427	gi|34538476	
*Clo2*	2BL	986	245	Bradi5g15417		
*Clo2*	2DL	877	245			
*Clo3*	6AS	907	217	Bradi1g70390		
*Clo3*	6BS	818	215			
*Clo3*	6DS	793	220			
*Clo4*	3AL	1039	217	Bradi1g70400		gi115467408
*Clo4*	3B	1075	217	Bradi1g44207		
*Clo4*	3DL	1084	217			
*Clo5*	6AL	1053	229	Bradi3g56810	gi|6900307	
*Clo5*	6BL	1051	224			
*Clo5*	6DL	1150	222			
*Clo6*	6AL	827	216	Bradi3g56820	gi|151420803	gi115467410
*Clo6*	6BL	735	205			
*Clo6*	6DL	815	214			
*Clo7*	7AS	827	198	Bradi1g44200	gi|151419867	gi115448521
*Clo7*	7BS	917	213			
*Clo7*	7DS	958	212			
*Clo8*	7AS	1102	214		gi|326515641	
*Clo8*	7BS	962	213			
*Clo8*	7DS	939	215			
*Clo9*	4AS	994	232	Bradi1g69571	gi|151426143	
*Clo9*	4BS	1129	235			
*Clo9*	4DL	1164	233			
*Clo10*	2AL	907	234		gi|326490092	gi115459382
*Clo10*	2BL	866	244			
*Clo10*	2DL	897	243			
*Clo10*	2DL	836	244			
*Clo11*	2AL	907	234		ViroBlast	
*Clo11*	2BL	1062	244			
*Clo11*	2DL	932	242			

^a^The full set of complete caleosin sequences for wheat and rye can be found in Additional file 16. The corrected Hv-*Clo6* cDNA of *H. vulgare* sequence and Hv-*Clo8* derived from ESTs and the Brachypodium sequence Bradi1g70400 with an extended CDS are included in Additional file 17.

^*b*^Caleosin sequences for Brachypodium, *H. vulgare*, and *O. sativa*, represent paralogous members of the gene family, and do not correspond to the individual homeologous gene copies found in wheat.

The accuracy of each contig sequence was judged to be excellent, as the contigs generally had a minimum depth of at least three ESTs and many had a depth of four to ten sequences. There was also a very good agreement between the contigs assembled from EST sequences, sequences of individual cDNA clones and the WSS assemblies, which indicates the high accuracy of the WSS database. Genes within homeologous groups had high nucleotide sequence similarity, ranging from 96% to 97% nucleotide sequence identity within the coding region. This high degree of similarity is common among homeologous copies of genes in wheat [35]. The WSS sequences are derived from shotgun 454 sequencing of chromosome arm specific BAC libraries, thus sequences have a chromosomal assignment, but not map location along the chromosomes. In nearly all cases, homeologous copies of genes were located on the same arm of homeologous chromosomes. One exception is *Clo9*. The A and D genome copies of *Clo9* are located on the short arm and long arm of chromosome 4A and 4D, which are considered homeologous, the B genome copy is located on the short arm of 4B which is not homeologous to the other two chromosome arms [36]. Ten of the 11 paralogous groups had three homeologous copies, but the caleosin 10 had four gene copies, one on each of the long arms of chromosome 2A and 2B and two copies identified on chromosome 2DL. These two copies of *Clo10* on 2DL had 93% nt sequence identity within the coding region, which is somewhat lower than the sequence identity among the homeologous copies on chromosomes 2A and 2B and one of the D copies, *Clo10-2D*.

The degree of similarity among the paralogous sequences of wheat spanned a wide range; the most similar paralogs were *Clo10* and *Clo11* which shared 89% nucleotide sequence identity and 95% amino acid sequence similarity within the conserved 174 EF-hand domain. The most dissimilar members of the wheat caleosins gene family were *Clo10* and *Clo4* which had 37% amino acid sequence identity and 57% similarity within the conserved domain. The size of the proteins encoded by members of the gene family ranged from 198 to 302 amino acids. All members of the gene family are characterized by the presence of a single EF-hand calcium binding region of approximately 174 amino acids and a predicted transmembrane domain of 20 amino acids.

### Caleosin genes in *Secale cereale*and *Hordeum vulgare*

**Figure 1:**
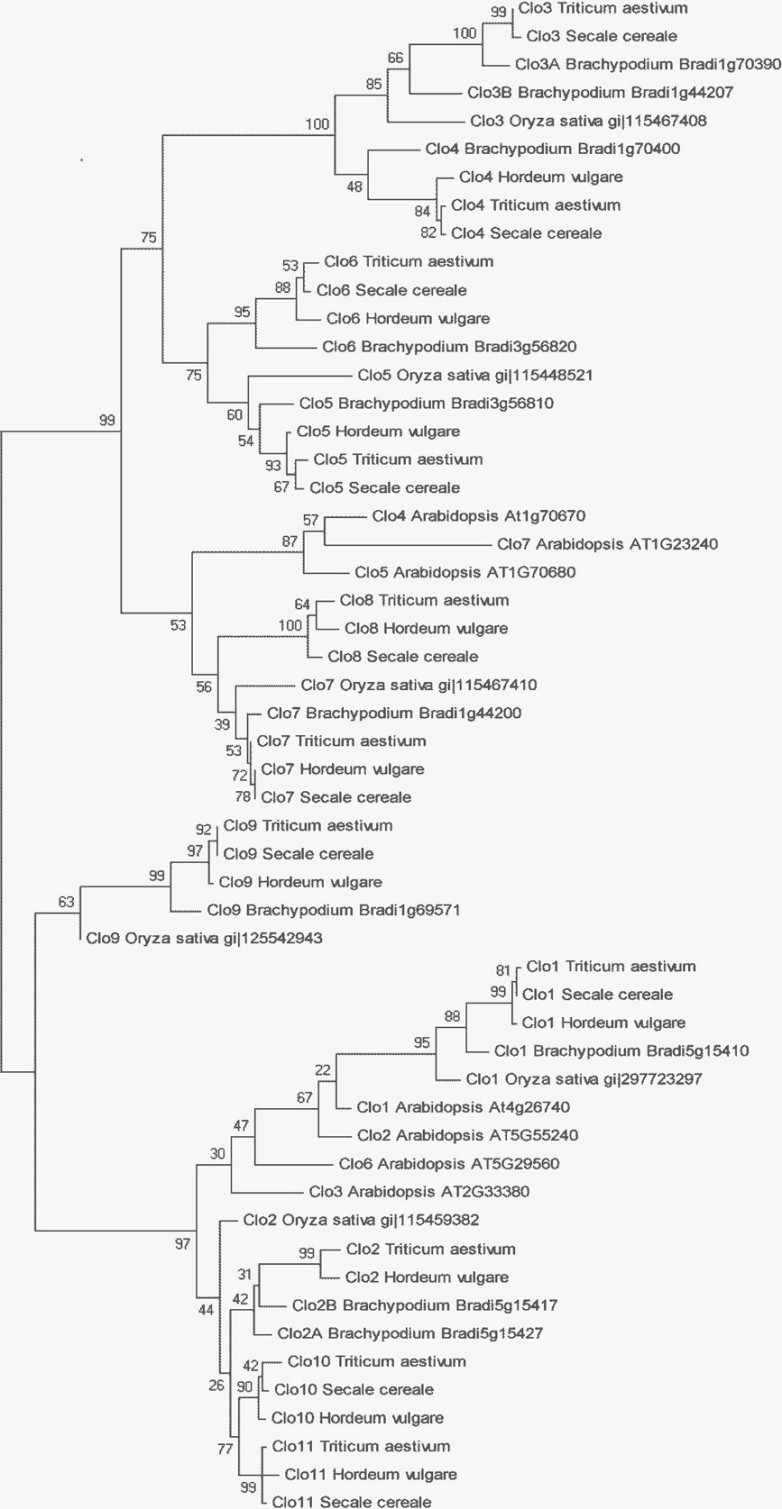
**Molecular phylogenetic analysis of the caleosin gene families of** ***T. aestivum*** **,** ***H. vulgare*** **,** ***B. distachyon*** **,** ***S. cereale*** **,** ***O. sativa*** **, and** ***A. thaliana*** **amino acid sequences.** The evolutionary history was inferred by using the maximum likelihood method based on the Whelan and Goldman model [21]. The tree with the highest log likelihood (−3638.1820) is shown. The tree is drawn to scale, with branch lengths measured in the number of substitutions per site. The values on the tree represent bootstrap confidence values inferred from 100 replicates. Brachypodium gene identifiers are taken from http://brachypodium.org/[13]; rice IDs are from GenBank and Arabidopsis IDs are from The Arabidopsis Information Resource (TAIR) [38]. Only one of each representative wheat caleosin homeologous groups was used in order to simplify the phylogenetic tree. The relationship among the wheat gene family members is shown in Additional file 5: Figure S2.

Ten full-length caleosin cDNA sequences in *S. cereale* (rye) were assembled from the Roche 454-cDNA sequence set. Only Caleosin 2 was not identified in our rye sequence set, though a 239 nt fragment of a gene with 97% sequence similarity to wheat Caleosin 2, is present in the whole genome shotgun sequence for rye [37]. This indicates that all 11 *Clo* genes are present in rye. The rye sequences showed between 90% and 99% sequence identity with their homologs in wheat within the coding region of each sequence. This high degree of similarity is common between wheat, barley, and rye, which are all members of the Triticeae tribe, as seen in the phylogenetic diagram in Figure 1.

Nine full length cDNA sequences of caleosin genes were identified in *H. vulgare*. Seven of these were obtained from the non-redundant database at GenBank, one sequence was assembled from ESTs, and one sequence, Hv-*Clo11*, was tentatively derived from second generation sequencing of the barley genomic sequence. The latter sequence could not be confirmed independently, but sequence differences between the other eight FL sequences from barley and the assembly1-morex rcba, suggest Hv-*Clo11* derived from the viroblast high through-put database may contain some inaccuracies. A *Clo3* ortholog was not identified in the barley sequence databases.

### Caleosin genes in *Brachypodium distachyon*

Ten caleosin genes were identified in *B. distachyon,* a species for which the complete genome sequence is available. Two of the sequences were modified by extending the ORF relative to the annotated sequence that is available for the *Brachypodium* genome. All contigs were verified as full-length by comparison to the wheat sequences; open reading frames and protein sequences were obtained.

### Conserved structural elements

The conserved domain in caleosins was identified by Batch CD Search Tool on NCBI and InterPro Scan for each paralogous group from *T. aestivum*, *H. vulgare*, and *B. distachyon*. Results were verified by comparing with the conservation score calculated by aligning multiple sequences with ClustalW2 [34]. The conserved EF-hand calcium binding domain of 174 amino acids is common to all caleosins. This EF-hand motif is composed of 36 amino acids with a calcium chelation loop and calcium ligating residues. The DGSLFE box which is the putative Casein Kinase Phosphorylation site is also highly conserved [2]. The alignment of all peptide sequences from *T. aestivum*, *H. vulgare*, and *B. distachyon* using ClustalW revealed the presence of other less conserved motifs, including the GS loop, transcription termination factor, and a trans-membrane domain.

### Intron-exon structure of caleosin genes in *T. aestivum*, *H. vulgare*, and Brachypodium

The 24 caleosin genes in *T. aestivum* for which full genomic sequences were available all contained 6 exons and 5 introns. Ten wheat caleosin genes for which partial coverage by the genomic contigs was available, showed the same intron-exon structure for the gene region that was covered. The details of intron and exon lengths for all available caleosins in *T. aestivum* are listed in Additional file 6: Table S3. Among the *H. vulgare* caleosin genes, five genes had six exons, one had four exons, one had five exons, and one had seven exons; two barley caleosins had only partial coverage by genomic contigs. In Brachypodium, seven caleosins had six exons, these include: Bradi5g15410, Bradi5g15417.1, Bradi5g15427.1, Bradi1g70390, Bradi1g44207, Bradi3g56820.1, Bradi1g44200, and Bradi1g69571 (Bradi5g15417.1 was miss-annotated as having 5 exons at the Gramene database); in contrast, Bradi3g56810 and Bradi1g70400 have 5 exons; however, these may have incomplete annotation. A summary of available caleosin gene exons for barley and Brachypodium can be found in Additional file 7: Table S4.

### Tissue-specificity of rye and triticale caleosin paralogs

**Figure 2:**
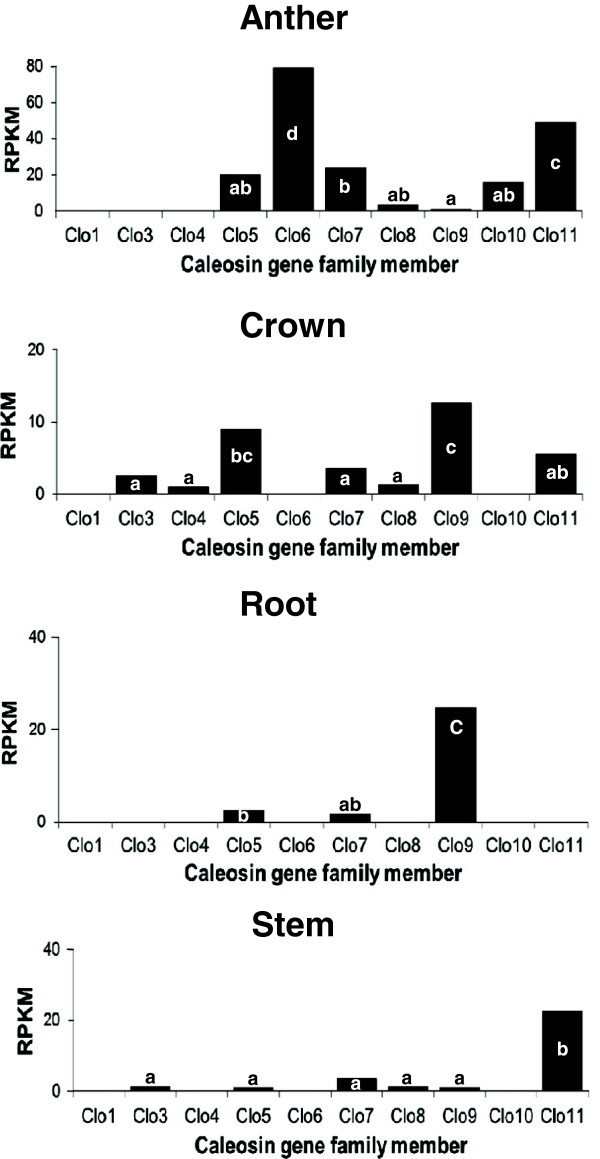
**The relative level of expression of ten caleosin gene family members measured in a panel of rye tissues.** The expression of caleosin gene family members was estimated in 454 cDNA sequence libraries from anther, crown, root and stem rye tissues using RNA-seq analysis. The aligned 454-cDNAs to each caleosin member were counted, then normalized based on gene lengths and library depths using the RPKM method. Values are the total RPKM of two replicates. A one-way ANOVA was carried out to test the significance of the differences in caleosin gene expression in each rye tissue. Duncan’s multiple range test was used to determine the significant differences in caleosin gene expression in each tissue. Rankings determined by Duncan’s test (p ≤ 0.05), are denoted by different letters, and are indicated on each bar in the graph.

**Figure 3:**
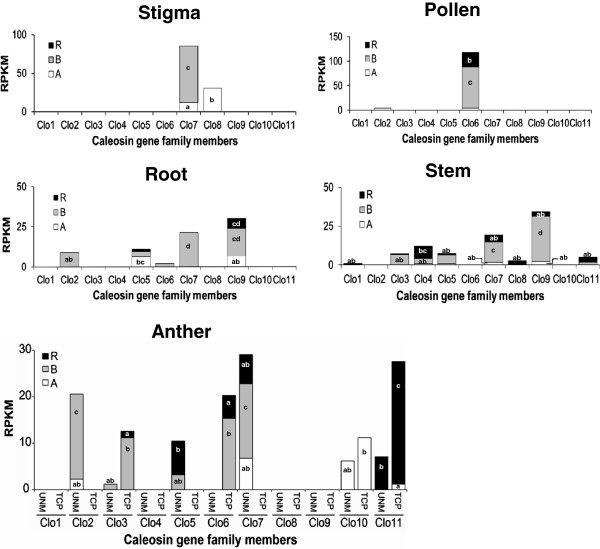
**The relative level of expression of eleven caleosin paralogs measured in a panel of triticale tissues.** The expression of thirty-two caleosin gene family members was individually measured in 454 cDNA sequence libraries from anther, pollen, root, stem, and stigma triticale tissues using RNA-seq analysis. The aligned 454-cDNAs to each caleosin member were counted, then normalized based on gene lengths and library depths using the RPKM method. The expression of each paralog is subdivided into expression of each of the three homeologs, visualized as black bars for the R subgenome, grey bars for the B subgenome, and white bars for the A subgenome. *Clo2* represented the expression of only the A and B homeologs. Values are the total RPKM of two replicates for stigma and pollen, two replicates each for UNM (uninucleate microspore) and TCP (tricellular pollen) anther tissues, and six replicates for stem. A one-way ANOVA was carried out to test the significance of global differences in caleosin gene expression in each triticale tissue. Duncan’s multiple range test was used to determine the significant differences in caleosin gene expression in each tissue. Rankings determined by Duncan’s test (p ≤ 0.05) are denoted by different letters, and are indicated on each bar in the graph. Bars not labeled are ranked as ‘a’. In stem tissue, the A, B, and R homeologues of *Clo5* and *Clo11* are all ranked as ‘ab’, and the A and B homeologues of *Clo4* are ranked as ‘ab’.

The abundance of ESTs for ten rye caleosin genes as well as twenty-two genes from eleven paralogous groups assigned to the A and B subgenomes in triticale was investigated to assess their relative expression level in more than 4.3 M and 7.1 M of 454 cleaned reads from four rye tissues (anther, crown, root, stem) and five triticale tissues (stigma, pollen, root, stem, anther), respectively. The data was normalized to take into account the sizes of the different tissue specific data sets and gene lengths, and is expressed as reads per kb of gene length per million 454-cDNA reads (RPKM), and presented in Figures 2 and 3, Additional file 8: Figure S3, and Additional file 9: Figure S4.

Caleosin gene family members were detected in all tissues sampled, and most tissues showed the expression of several paralogous members of the gene family. Expression of three caleosin members (*Clo5, Clo7, and Clo9*) were detected in rye root tissue, with the expression of *Clo9* being dominant, and *Clo5* and *Clo7* detected at very low levels (Figure 2). Most caleosin transcripts were detected in multiple tissues; however, *Clo6* and *Clo10* were only detected in anther tissue, with *Clo6* detected at relatively high levels (Figure 2 and Additional file 8: Figure S3), demonstrating high tissue specificity for these caleosin members. In contrast, *Clo5* and *Clo7* were detected in all four tissues (Additional file 8: Figure S3).

Triticale showed a similar diversity of expression of all gene family members. Among the five tissue types included in the analysis, anther tissue was sampled at two stages of development: UNM (uninucleate microspore) and TCP (tri-cellular pollen). A two-way ANOVA showed significant differences between expression of caleosin genes (p = 0.000), and also a significant interaction effect between gene and developmental stage (p = 0.000), indicating that there is a different level of caleosin gene expression at different stages of anther development. Expression of only two caleosin genes were detected in triticale pollen (*Clo2,* and *Clo6*), and stigma tissues (*Clo7* and *Clo8*), and expression of five caleosin genes (*Clo2, Clo5*, *Clo6, Clo7* and *Clo9*) were detected in root tissue, with *Clo7* and *Clo9* expression being dominant (Figure 3). *Clo7* and *Clo9* expression was also dominant in stem tissue, and *Clo7* was found to show a broad tissue expression pattern, as it was detected in four of the tissues investigated (Additional file 9: Figure S4). In anthers, *Clo7* showed high expression specifically at the UNM stage, whereas it was not detected at the TCP stage. Conversely, *Clo11* was observed to be primarily expressed at the TCP stage, and to a lesser extent at the UNM stage of anther development (Figure 3). Therefore, there is tissue specificity for certain caleosin members, as was observed with expression in rye tissues. Overall, these results demonstrate a very diverse pattern of tissue-specific expression of the caleosin genes.

### Tissue-specificity of wheat and barley caleosin paralogs

Tissue-specificity of caleosin gene family members was also analyzed using independent data sets obtained from the PLEXdb database [31], and compared to the results for rye and triticale described above. Eight of the *T. aestivum* caleosins are represented on the wheat 61 K Affymetrix microarray, similarly nine of the barley caleosins are represented on the 22 K Affymetrix microarray. Gene expression in multiple tissues and stages of development was assayed in *T. aestivum* by Schreiber et al. [29], and in barley by Druka et al. [30]. The wheat data shows a diversity of expression of caleosin family members in the tissues assayed, and appears to partially corroborate rye and triticale expression results (Additional file 10: Table S5). The wheat microarray data reveals that the *Clo9* paralog is relatively highly expressed in root tissue (Additional file 10: Table S5), as was found in rye and triticale expression data described above. The wheat microarray data also reveals relatively high expression of the *Clo7* paralog across tissues, in agreement with the expression data for triticale. Both *Clo6* and *Clo7* paralogs showed relatively high expression in anther tissue in the wheat microarray data, (Additional file 10: Table S5) which was similar to the observations in the 454 sequence data from both rye and triticale, although *Clo7* was expressed significantly less than *Clo6* in rye anther (Figures 2 and 3). In contrast, *Clo11* was seen to be highly expressed in anther tissue of rye and triticale (Figures 2 and 3), but not in the wheat anthers assayed by microarray analysis (Additional file 10: Table S5). Such differences in expression may be accounted for by differences in experimental design, stages of development, or may reflect species differences.

The barley microarray data reveals that both *Clo7 and Clo9* paralogs are relatively highly expressed in root tissue (Additional file 11: Table S6), which is in agreement with the 454 expression data for triticale (Figure 3). *Clo9* was also highly expressed in rye roots, but *Clo7* was expressed at significantly lower levels in the same tissue (Figure 2). *Clo8* was also seen to be expressed at high levels in the roots of barley in the microarray data, but not in the roots of rye and triticale in the 454 sequence sets. *Clo6* and *Clo10* are highly expressed in barley anther tissue as well as in the anthers of both rye and triticale; however, 454 data for rye and triticale shows high expression of additional family members in anther tissue (Additional file 11: Table S6, Figures 2 and 3). *Clo4* showed no significant expression in any of the tissues assayed in the barley microarray data which parallels results from rye and triticale (Additional file 11: Table S6, Figures 2 and 3). Some differences in expression between the barley microarray data and the rye and triticale 454 datasets include high levels of *Clo8* in the crown of barley but not of rye (Additional file 11: Table S6, Figure 2). In addition there were differences in gene expression between the microarray data of wheat and barley, for example, several caleosins showed significant expression in wheat pistils but not in the same tissue in barley. Overall, the wheat and barley array data show a diverse pattern of caleosin gene expression in various tissues, and represent independent data sets that corroborate some of the tissue-specific caleosin gene expression patterns observed for rye and triticale.

### The expression of caleosins under stress conditions

Stress response experiments offer insight into changes in gene expression in response to environmental stresses. In response to osmotic stress administered as PEG treatment, *Clo3B*, *Clo5B, Clo5R,* and *Clo11R* were observed to be significantly reduced compared to the level of the control in osmotically stressed seedlings (Additional file 12: Table S7)*. Clo9* was seen to be induced about four fold in the roots of salt-stressed rye plants (Additional file 13: Table S8). In contrast, salt stress was not seen to alter the expression of caleosins in the roots of similarly treated triticale plants. Previously published microarray analysis also sheds light on the involvement of caleosins in the stress response. The microarray analysis of cold acclimation in wheat by Monroy et al. [32] included probes for four caleosin family members, *Clo2* to *Clo5*, and results show *Clo3* to be strongly induced in shoots by cold treatment of 4°C. *Clo3* was induced within 6 h of treatment in winter wheat and maintained increased levels of expression up to 14 days of cold acclimation. *Clo3* was also induced by cold treatment in spring wheat but showed a different profile of induction as seen by a significant G X T effect in a 2-way ANOVA (Additional file 14: Table S9). Two other microarray analyses looked at the expression of caleosin family members under stress conditions. One study by Aprile et al. [33] looked at the effect of drought stress on *Triticum* caleosin expression, and although this data set showed small changes in expression in response to stress, they were found to be statistically non-significant. Another study looked at the ABA response in barley with the 22 k Affymetrix microarray [31; GEO Accession: GSE10328], and results showed that both *Clo10* and *Clo11* had significant increases in expression due to ABA stress (Additional file 15: Table S10).

### The effect of polyploidization on the expression of R subgenome caleosins

**Figure 4:**
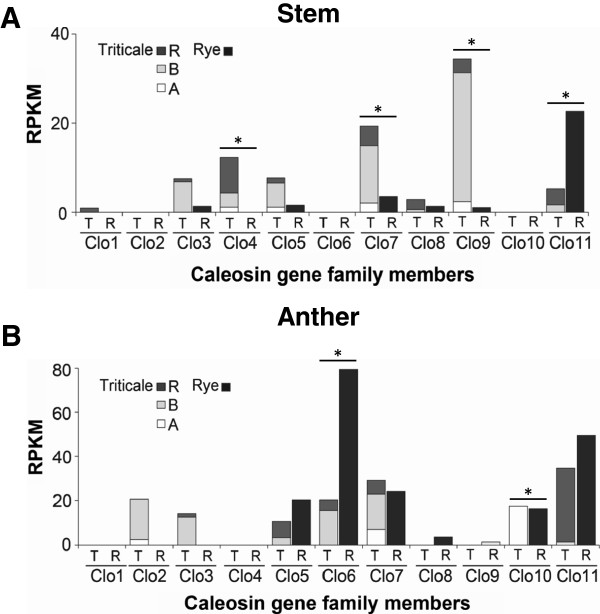
**A comparison of** ***Clo*** **gene expression in rye and triticale, to assay the changes in gene expression as a result of polyploidization.** The expression of caleosin gene family members was measured using RNA-seq analysis in the stem **(A)**, and anther **(B)**, of rye and triticale, respectively. The 454-cDNA sequences aligned to each caleosin member were counted, then normalized based on gene lengths and library depths using the RPKM method. The expression of each paralog is subdivided into expression of each of the three homeologs for triticale (T), visualized as black bars for the R subgenome, grey bars for the B subgenome, and white bars for the A subgenome. The expression of caleosin family members in rye (R) is represented by solid black bars. χ2 contingency tests based on the null hypothesis that the level of individual R genome caleosins in triticale was not different than one third of the level of expression of the genes in rye were carried out. The *marks individual caleosins with p < 0.05, where the null hypothesis was not accepted.

Two tissue types, stem and anther, were sampled for both rye, and triticale. This facilitated the comparison of the R genome homeologs in both the rye and triticale genetic background, and examination of the effect of polyploidization on the expression of R gene copies. In stem tissue, the most striking differences between the two species was *Clo11-R*, which was the most highly expressed caleosin in rye stems, but was expressed at low levels in triticale as were the A and B genome copies of *Clo11*(Figure 4A). *Clo4-R* was not detected in rye stem tissue but was expressed in moderate levels in triticale stems (Figure 4A), thus *Clo11-R* appears to have been suppressed and *Clo4-R* appears to have been activated by the polyploidization event. In anthers, the relative level of expression of most caleosin gene R homeologs was lower in triticale than in rye, and some rye genes showed especially marked differences in expression in the two species. *Clo10-R* and *Clo6-R* were highly expressed in rye, whereas *Clo10-R* was not detected in triticale, indicating a loss of expression in the R genome of triticale in anthers (Figure 4B). *Clo6-R* also had very low levels of expression in triticale (Figure 4B). *Clo8-R* was detected in rye anthers but not in triticale anthers (Figure 4B); however, the level of expression was relatively low in rye, thus loss of expression in triticale could not be assessed with confidence.

## Discussion

### The caleosin gene family

The 11 paralogous groups of caleosin genes present in *T. aestivum* represents the largest gene family for caleosins among the seven species included in this analysis. Though the entire wheat genome has not yet been completely sequenced, the description of the caleosin gene family appears to be complete or nearly so, since orthologs for all caleosins detected in rye, barley and Brachypodium were also found in wheat. In addition, three homeologous copies for each paralogous group were detected in wheat as well as a fourth member of the *Clo10* group. All 32 of the wheat caleosin genes identified in the EST databases at GenBank were also identified in the WSS and two additional genes, *Clo4-B* and *Clo11-A*, that were not represented in the *T. aestivum* EST database at GenBank were identified in the WSS. This indicates that the depth of the WSS is very comprehensive. The wheat caleosin genes were also represented in the whole genome sequencing database (Genbank Whole-genome shotgun contigs database) [39]. Brachypodium, the most closely related monocot species with a completed and annotated genome sequence available, has seven caleosin genes and rice has six caleosin genes. All caleosin genes in rice and Brachypodium have orthologous sequences in wheat, barley and rye, as judged by sequence similarity, and seen in the phylogram in Figure 1. Since these more distantly related species have gene family members which share branches with sequences from wheat throughout the tree, it seems that the representation of caleosins from wheat is likely complete. The larger number of caleosin genes in Triticeae, supported by identified sequences from wheat, rye and barley, and the representation of genes from *Oryza* on the major sub-branches in the phylogram indicate that the Triticeae had five gene duplications after the evolutionary separation from the *Oryza,* and before the separation of the three wheat progenitor species from each other. These duplications in the Triticeae lineage after the separation from rice are the *Clo3* and *Clo4* duplication, the *Clo5* and *Clo6* duplication, the *Clo7* and *Clo8* duplication, and the *Clo2* and *Clo10* duplication, and subsequently the *Clo10* and *Clo11* duplication. The localization of these putative gene duplications on the same chromosomes supports this notion, since gene duplications often occur as tandem duplications [40], though further investigation would be required to demonstrate that the duplications were in tandem. Hv-*Clo3* was not identified in the barley data sets in GenBank; this may represent gene loss, loss of gene expression or may be due to incomplete transcriptome or genome sequence from these species in the current databases. The lack of a full length sequence for the rye *Clo2*, but the presence of a gene sequence fragment for it in a high throughput sequence database is indicative of the advanced but still incomplete state of the sequencing for these species. The length of the branches in Figure 1, are scaled according to amino acid differences between sequences, thus providing an estimate of evolutionary distance. The duplication of *Clo10* and *Clo11* appear to have happened more recently than other duplications, and the presence of *Clo10* and *Clo11* only in wheat, rye and barley provides supporting evidence for duplication only after the separation from the *Brachypodium lineage*. A similar evolutionary pattern is also observed for *Clo5* and *Clo6*, though that duplication appears to be older than the *Clo10*, *Clo11* duplication.

In Brachypodium, Bd-*Clo1* and two copies of *Clo2* (Bd-*Clo2-A*, and Bd-*Clo2-B*) ([13]; Bradi5g15410, Bradi5g15417, and Bradi5g15427) are identified as tandem duplications. In addition, Bd-*Clo3-A* (Bradi1g70390) and Bd-*Clo4* (Bradi1g70400) are also adjacent to each other. However *Clo3-A* and *Clo3-B* which share high sequence similarity and are both on chromosome 1, are not closely linked. Brachypodium provides evidence for both recent and old tandem duplications for caleosins as well as relatively recent non-tandem duplications. Arabidopsis’ caleosin genes are largely clustered together in the phylogenetic tree suggesting that gene duplication events occurred independently in the monocotyledonous and dicotyledonous branches of the tree.

### Variation of caleosin gene expression in plant tissues

The diverse expression profile of the caleosin genes in rye and triticale tissues, as well as in wheat and barley tissues, suggests that these calcium binding proteins likely play a broad role during plant development. Whereas some caleosins exhibited more restricted, tissue-specific patterns of expression, such as *Clo6* and *Clo10*, others such as *Clo7*, were detected in almost all rye and triticale tissues sampled. It is therefore conceivable that some caleosins may have a more general, ‘housekeeping’ role in most plant tissues and cell types, whereas other caleosins may have tissue- and cell-type specific roles in signaling and regulation during plant development. Evidence that *Clo3* in wheat acts as a GAP for the α-subunit of the heterotrimeric G protein [1], raises the possibility that members of the gene family may play key roles in signaling. The expression of other calcium-binding proteins in plants that function in all cell types or that have more restricted, tissue-specific functions, have been previously reported. For example, calmodulin, the predominant Ca^2+^ sensor, plays a critical role in decoding Ca^2+^ signatures into proper cellular responses in numerous tissue types and cellular compartments in eukaryotes [41]. Six members of the calmodulin-like protein gene family are expressed in a developmentally controlled pattern during nodulation in the roots of *Medicago truncatula*[42], and a kinesin-like calmodulin-binding protein, was found to be selectively expressed in the flowers, roots, and leaves of Arabidopsis [43].

Although *T. aestivum Clo3* was found to be expressed in several triticale and rye tissues, there was no expression in several tissues analyzed. Although these results are in agreement with previous work [2], demonstrating that the expression of At-*Clo3*, the ortholog of Ta-*Clo3*, was undetectable in Arabidopsis root using northern analysis, we have detected expression in Arabidopsis in response to abscisic acid treatment [unpublished observations] using transgenic plants with promoter:Gus gene constructs. This underscores the challenges of tissue-specific expression analysis, since expression can be regulated developmentally, as well as by other factors such as environmental conditions and hormonal fluxes, and may not be identified in the tissue samples taken at a limited number of time points of development that are represented in the cDNA sequence databases. The induction and repression of several caleosin gene family members in response to salt stress, cold acclimation and osmotic stress implies a wide role for members of the gene family in the environmental stress response. These observations warrant a more in-depth analysis of the tissue specificity and the time course for the changes in gene expression. Further study of potential partners in protein-protein interaction is also warranted, since *Clo3* of wheat has previously been shown to interact with both the α-subunit of the heterotrimeric G protein complex as well as members of the PI-PLC gene family [1].

Interestingly, the gene expression results demonstrate the effect of polyploidization on the expression of R subgenome caleosins. The synthetic triticale is an intergeneric allohexaploid generated from *Triticum durum* and rye. The initial combination of two or more genomes in one organism may lead to considerable genomic reorganization and changes in gene expression relative to the parental species. Soon after polyploidization, triticale underwent a loss of its combined DNA content in the range of 22 to 30% [44, 45], and DNA elimination of repetitive DNA and low-copy sequences from the rye genome in triticale have been reported in molecular studies [46, 47]. The effect of polyploidization was clearly observed in the case of several caleosins, such as the expression of the rye homeologs *Clo6-R* and *Clo10-R*, in triticale. The rye *Clo6-R* and *Clo10-R* homeologs were found to be suppressed in anthers, although expression of these caleosins were relatively high in the corresponding rye tissues. Tissue-specific silencing of homeologs from one of the genomes of polyploid species has been reported in other allopolyploids including *Tragopogon miscellus*[48], and *Gossypium hirsutum*[49], though the mechanisms that lead to suppression are somewhat speculative at this point. Since these genes are expressed in other triticale tissues, gene deletion is clearly not the explanation for suppression, and mechanisms related to chromatin remodelling or the incompatibility of signaling and regulation pathways of parental genomes in newly derived polyploids warrants investigation.

## Conclusions

An apparent full set of wheat caleosin gene sequences were acquired as full length cDNAs, the open reading frames were identified, and the peptide sequences were obtained. The gene sequences were confirmed with the WSS database. One member of the caleosin gene family, *Clo3*, has previously been identified as a stress inducible gene encoding a Ca^2+^ binding protein that acts as a negative regulator of the α subunit of the heterotrimeric G protein GA3 [1]. The identification and full description of the gene family for caleosins can be a significant step in further investigating the role of members of this gene family in signaling and regulation. The very diverse pattern of tissue-specific expression indicates a potential for a very broad role in signaling and regulation throughout plant development.

## Availability of supporting data

The full set of full length caleosin cDNA sequences for wheat and rye can be found in Additional file 16. The corrected Hv-*Clo6* cDNA of *H. vulgare* sequence and Hv-*Clo8* derived from ESTs and the Brachypodium sequence Bradi1g70400 with an extended CDS are included in Additional file 17. In addition, thirty one wheat caleosin sequences are being deposited at GenBank; seven of these are derived from direct sequencing of cDNA clones and have been deposited in the nr database with accession numbers HQ020505 and KJ523887 to KJ523892; 25 of these are derived from assembly of EST sequences and are being deposited at GenBank as Third Party Annotations (TPA). Five sequences are derived from triticale 454 EST libraries which are identical to *T. aestivum* caleosins and are being submitted to the Transcriptome Shotgun Assembly (TSA) database. Three wheat caleosin mRNA (i.e. cDNA) equivalent sequences Ta-*Clo4-B*, Ta-*Clo10-2-D*, Ta-*Clo11-A* were derived from genomic sequences at the WSS database and do not qualify for deposit at GenBank. The ten *Secale cereale* caleosin sequences derived from 454 cDNA sequences are being deposited in the TSA database at GenBank. The raw *Secale cereale* 454 sequences have been deposited at the DNA Data Bank of Japan with identifier DRA000384.

## Electronic supplementary material

Additional file 1: Table S1: Summary of rye cDNA libraries and derived 454 reads. (XLSX 11 KB)

Additional file 2: Table S2: Summary of triticale cDNA libraries and derived 454 reads. (XLSX 14 KB)

Additional file 3: Figure S1: Workflow used to measure the abundance of caleosin gene family members in thirteen rye and triticale 454-cDNA libraries expressed in different tissues. (PDF 75 KB)

Additional file 4: **Identifiers for** ***Clo*** **genes on microarrays.** (PDF 14 KB)

Additional file 5: Figure S2: Molecular phylogenetic analysis of *T. aestivum* caleosin nucleotide sequences by the maximum likelihood method. (PDF 36 KB)

Additional file 6: Table S3: Intron-exon structure of caleosin genes in *T. aestivum*. (XLSX 15 KB)

Additional file 7: Table S4: Caleosin gene exons in *H. vulgare* and Brachypodium. (XLSX 12 KB)

Additional file 8: Figure S3: The relative level of expression of ten caleosin gene family members in four different rye tissues based on 454 sequencing. The data in this figure is a reorganized version of the data presented in Figure 2. (PDF 83 KB)

Additional file 9: Figure S4: The relative level of expression of eleven caleosin gene family members in five different triticale tissues. The data in this figure is a reorganized version of the data presented in Figure 3. (PDF 105 KB)

Additional file 10: Table S5: Microarray analysis of caleosin gene expression measured in a panel of thirteen *Triticum aestivum* tissues. (XLSX 15 KB)

Additional file 11: Table S6: Microarray analysis of caleosin gene expression measured in a panel of fifteen *Hordeum vulgare* genotype Morex tissues. (XLSX 15 KB)

Additional file 12: Table S7: Caleosin expression in response to PEG treatment of triticale seedlings measured in 454 cDNA libraries. (XLSX 12 KB)

Additional file 13: Table S8: Comparison of caleosin expression in salt treated and control rye roots (RPKPM) measured in 454 cDNA libraries. (XLSX 16 KB)

Additional file 14: Table S9: Microarray analysis of the effect of cold treatment on caleosin gene expression in wheat. (XLSX 16 KB)

Additional file 15: Table S10: Microarray analysis of levels of expression of the caleosin paralogs in barley crown tissue under ABA treatment. (XLSX 12 KB)

Additional file 16: **The complete set of full length cDNA/mRNA sequences for caleosins from wheat and rye.** (PDF 109 KB)

Additional file 17: **Corrected caleosin sequences from** ***Hordeum vulgare,*** **and** ***Brachypodium distachyon.*** These versions of the sequences are not available in GenBank. (PDF 218 KB)

## Abbreviations

GAP: GTPase activating protein
PI-PLC: Phosphoinositide-specific phospholipase C
RD20: Responsive to dehydration 20
RPKM: Reads per kb of gene length per million reads
UNM: Uninucleate microspore
TCP: Tricellular pollen
PEG: Polyethylene glycerol.
