# Supplementary material for: Characterization of the caleosin gene family in the Triticeae
Source: BMC Genomics. 2014 Mar 27;15(1):239. doi: 10.1186/1471-2164-15-239 (PMC3986672; doi:10.1186/1471-2164-15-239)
Supplement: Supplementary file 3 — Additional file 3: Figure S1: Workflow used to measure the abundance of caleosin gene family members in thirteen rye and triticale 454-cDNA libraries expressed in different tissues. (PDF 75 KB) [file 12864_2013_7045_MOESM3_ESM.pdf]

Additional file 3

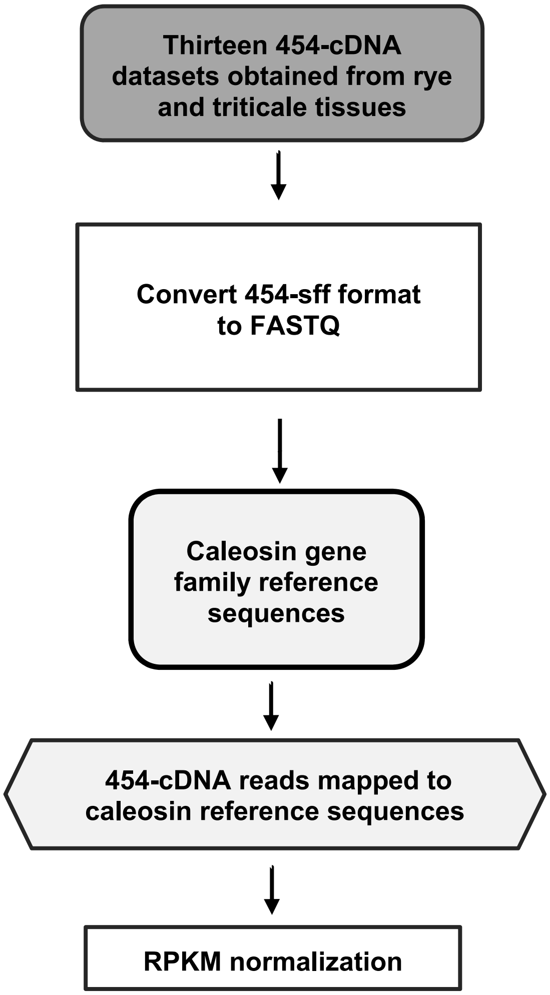

Figure S1. Workflow used to measure the abundance of caleosin gene family members in rye and triticale 454-cDNA libraries expressed in different tissues. 454-cDNA reads were converted from 454-sff format to FASTQ format through Galaxy server [28]. High quality rye and triticale 454-cDNA reads obtained from different tissues were aligned to caleosin gene family FL-cDNAs using CD-HIT-EST-2D [29]. The expression of each gene was normalized using RPKM normalization method.
