# Supplementary material for: Characterization of the caleosin gene family in the Triticeae
Source: BMC Genomics. 2014 Mar 27;15(1):239. doi: 10.1186/1471-2164-15-239 (PMC3986672; doi:10.1186/1471-2164-15-239)
Supplement: Supplementary file 4 — Additional file 4: Identifiers for Clo genes on microarrays. (PDF 14 KB) [file 12864_2013_7045_MOESM4_ESM.pdf]

Additional file 4

Identifiers for *Clo* genes on Microarrays

Wheat, *Triticum aestivum*
Identifiers for *Clo* genes on the Monroy *et al*. (2007) wheat microarray

Clo2 Tr004_D02
Clo3 Tr002_P09 and Tr016_E17
Clo4 Tr014_M12
Clo5 Tr007_O04

Identifiers for *Clo* genes on the *Triticum aestivum* 61K Affymetrix microarray

Clo1 Ta.2720.1.S1_at
Clo2 Ta.6018.1.S1_at
Clo3 not present
Clo4 TaAffx.1074.1.S1_at
Clo5 Ta.8740.1.S1_at
Clo6 TaAffx.79797.1.S1_at
Clo7 Ta.14471.1.S1_a_at
Clo8 TaAffx.71531.2.S1_at
Clo9 Ta.7279.1.S1_at
Clo10 not present
Clo11 Ta.9830.1.A1_at

Barley, *Hordeum vulgare*,
Identifiers for *Clo* genes on the *Hordeum vulgare* 22K Affymetrix microarray

Clo1	Barley1_03343
Clo2	Barley1_04461
Clo3	not present
Clo4	Barley1_26711
Clo5	Barley1_04442
Clo6	Barley1_03393
Clo7	Barley1_07208
Clo8	Barley1_07980
Clo9	Barley1_07208
Clo10	Barley1_49624
Clo11	not present
