# Supplementary material for: Characterization of the caleosin gene family in the Triticeae
Source: BMC Genomics. 2014 Mar 27;15(1):239. doi: 10.1186/1471-2164-15-239 (PMC3986672; doi:10.1186/1471-2164-15-239)
Supplement: Supplementary file 5 — Additional file 5: Figure S2: Molecular phylogenetic analysis of T. aestivum caleosin nucleotide sequences by the maximum likelihood method. (PDF 36 KB) [file 12864_2013_7045_MOESM5_ESM.pdf]

Additional file 5

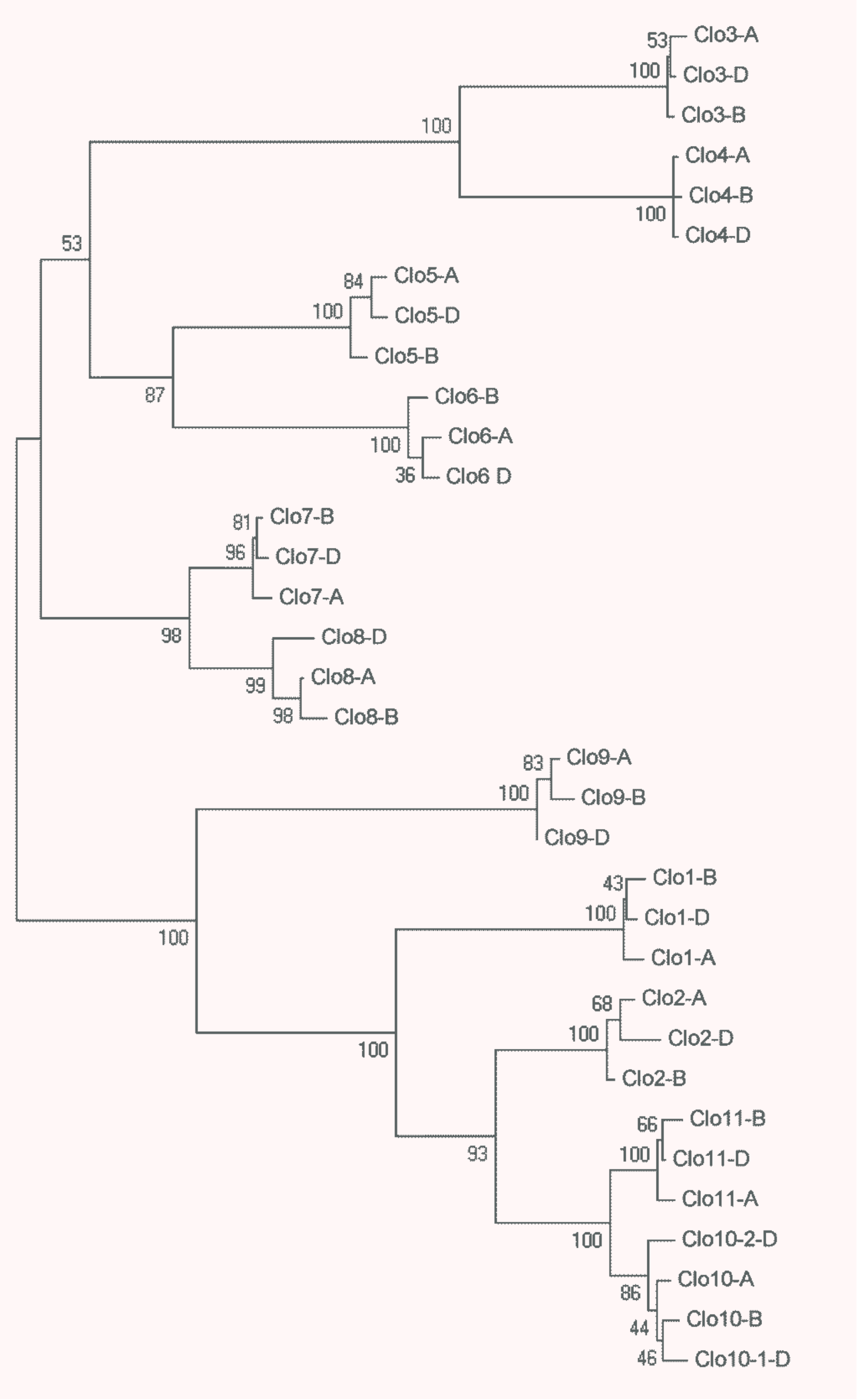

Figure S2. Molecular phylogenetic analysis of *T. aestivum* caleosin nucleotide sequences by maximum likelihood method. The evolutionary history was inferred by using the maximum likelihood method based on the Jukes-Cantor model [20]. The tree with the highest log likelihood (-6347.7079) is shown. The tree is drawn to scale, with branch lengths measured in the number of substitutions per site. The values on the tree represent bootstrap confidence values inferred from 100 replicates.
