# Supplementary material for: Characterization of the caleosin gene family in the Triticeae
Source: BMC Genomics. 2014 Mar 27;15(1):239. doi: 10.1186/1471-2164-15-239 (PMC3986672; doi:10.1186/1471-2164-15-239)
Supplement: Supplementary file 8 — Additional file 8: Figure S3: The relative level of expression of ten caleosin gene family members in four different rye tissues based on 454 sequencing. The data in this figure is a reorganized version of the data presented in Figure 2. (PDF 83 KB) [file 12864_2013_7045_MOESM8_ESM.pdf]

Additional file 8

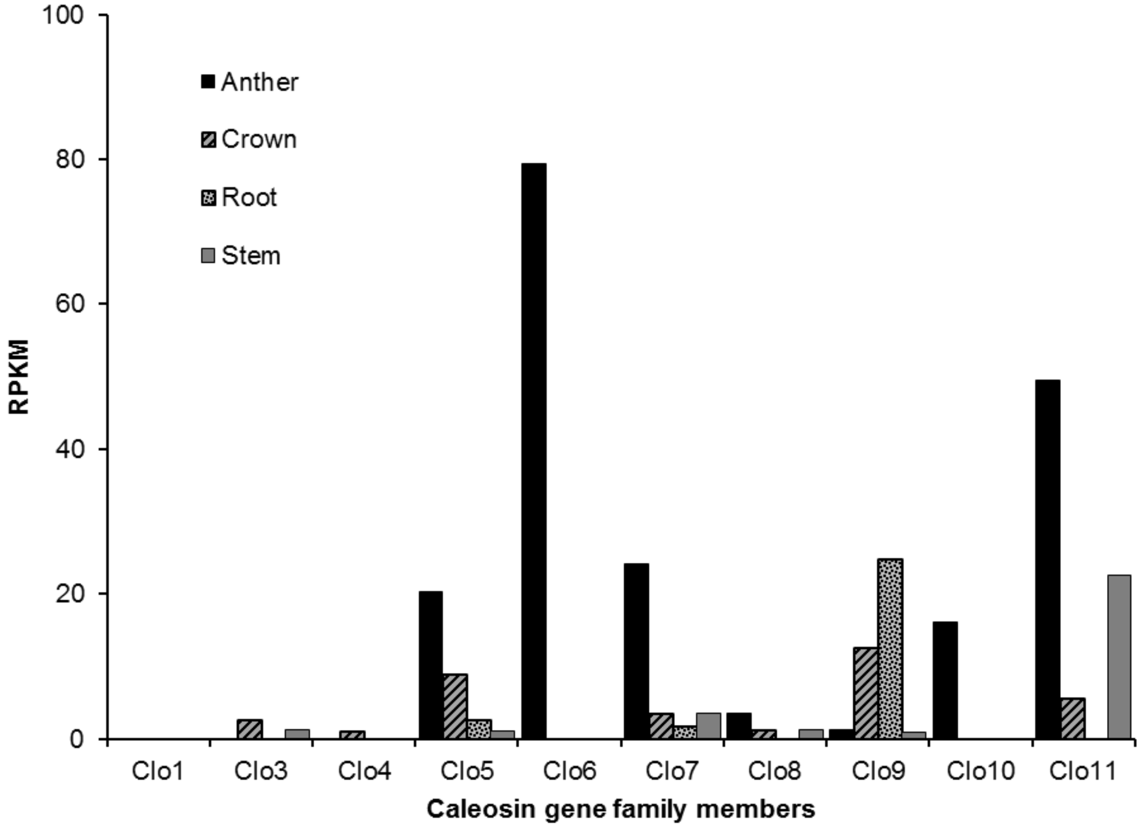

Figure S3. The relative level of expression of ten caleosin gene family members in four different rye tissues based on 454 sequencing. The data in this figure is a reorganized version of the data presented in Figure 2. The expression of caleosin gene family members was estimated in anther, crown, root and stem rye tissues using RNA-seq analysis. The aligned 454-cDNAs to each caleosin member were counted, then normalized based on gene lengths and library depths using the RPKM method.
