# Supplementary material for: Characterization of the caleosin gene family in the Triticeae
Source: BMC Genomics. 2014 Mar 27;15(1):239. doi: 10.1186/1471-2164-15-239 (PMC3986672; doi:10.1186/1471-2164-15-239)
Supplement: Supplementary file 9 — Additional file 9: Figure S4: The relative level of expression of eleven caleosin gene family members in five different triticale tissues. The data in this figure is a reorganized version of the data presented in Figure 3. (PDF 105 KB) [file 12864_2013_7045_MOESM9_ESM.pdf]

Additional file 9

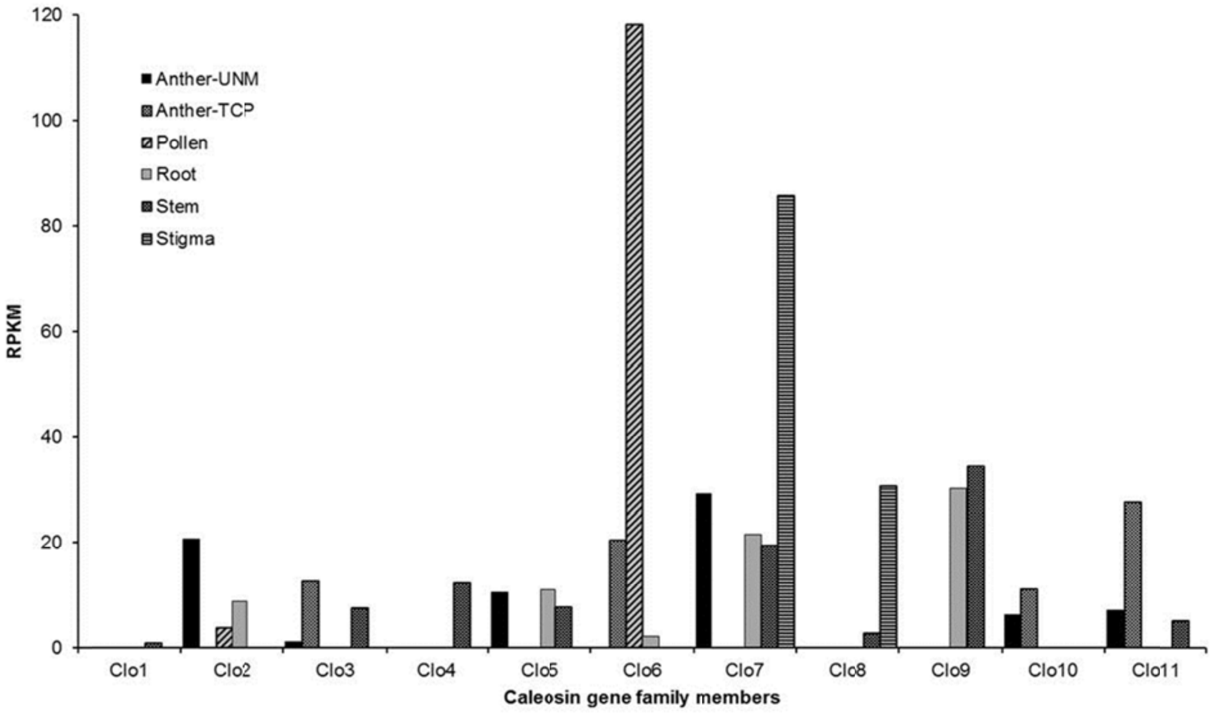

Figure S4. The relative level of expression of eleven caleosin gene family members in five different triticale tissues. The data in this figure is a reorganized version of the data presented in Figure 3. The expression of thirty-two caleosin gene family members was individually measured in anther, pollen, root, stem and stigma triticale tissues using RNA-seq analysis. Anther tissue was assayed at two different stages of development: UNM (uninucleate microspore) and TCP (tricellular pollen). The aligned 454-cDNAs to each caleosin member were counted, then normalized based on gene lengths and library depths using the RPKM method. The total expression of the three homeologs was combined to graph the expression of each paralogous set, except Clo2 which represents the expression of only the A and B homeologs.
