# Supplementary material for: Characterization of the caleosin gene family in the Triticeae
Source: BMC Genomics. 2014 Mar 27;15(1):239. doi: 10.1186/1471-2164-15-239 (PMC3986672; doi:10.1186/1471-2164-15-239)
Supplement: Supplementary file 16 — Additional file 16: The complete set of full length cDNA/mRNA sequences for caleosins from wheat and rye. (PDF 109 KB) [file 12864_2013_7045_MOESM16_ESM.pdf]

Additional file 16

Full set of caleosin sequences from wheat (*T. aestivum*) and rye (*S. cereale*)

The sequences below are the complete collection of full length cDNA/mRNA sequences for caleosins from wheat and rye. Sequences derived from cDNA clones are being submitted to GB. Others derived solely from GB ESTs may not be eligible for submission to GB as third party annotations. Sequences derived from Triticale 454 EST libraries which are identical to *T. aestivum* caleosins are eligible for submission and this is in progress.

1) Wheat (*T. aestivum*)

```
>Clo1-A [organism=Triticum aestivum] EF-hand calcium binding protein,
caleosin mRNA, full CDS
CCACGCGTACGCTCGGTAGCAGAAGCAGCACTCCACAGCTTGCCGGCAAGTTATCTTCTC
CACGATTCCGCTCACGGTTCATCGGTTGCCACCCGGTTCTGGTGCGCCGTCGCAGAGCGG
CGGAGATGGCCACCAAGGGGCGCAAGGTCGTCGAGGTCCGTGACGCGAGCAGGACGGACG
GGAAGGGCGACGCCGCGGACGTCCACGTCGTCCGCGAGGGGAAGAGCGACTACGACACCG
CCGGCGGCGTCGGCCACCGTGGCGCGAACAAGACGGATGGGCATGGCGACGCGGGCAGCA
TGGTCGGGGACATCCGGGGCATCGACGGGAAGGACTCCCTGAAGATCGTCGCGATGCAGG
CGCCGGTCACCGTGGAGCGCCCCGTCCGCGGCGACCTCGAGGAGCACATCCCCAAGCCCT
ACTTGGCGAGGGCTCTGGCGGCGCCGGACATGTACCACCCGGAGGGGACGACGGATGATC
ACCAGCACCACAACATGAGCGTGCTGCAGCAGCACGTCGCCTTCTTCGACCGCGACAACA
ACGGCATCATCTATCCCTGGGAGACTTACGATGGGTGCCGTGCGGTTGGGTTCAACGTGT
TCACGTCCGCCTTCATCGCGTTTCTGGTGAACCTGGTCATGAGCTATCCCACCCTGCCCG
GCTGGCTGCCGAACCCCCTCTTCCCGATCTACGTGCACAACATCCACAAGAGCAAGCACG
GGAGTGACTCGGGGACCTACGACAAGGAGGGCCGGTTCATGCCGGTGAACTTCGAGAACA
TCTTCAGCAAGTACGCCCGCACGTACCCGGACAGGCTGAGCTACAGGGAGCTGTGGCGGA
TGACGGAGGGGTCCCGGGAGGTGTTCGACTTCGTCGGCTGGGTGGCGATGAAGCTGGAGT
GGTCGTTCTTGTACGTGCTGGCTCGGGACGACGAAGGGTACCTGTCGAGGGAGGCCATCC
GGCGGATGTACGACGGCAGCCTCTTCGAGTACATGGAGAGGCAGCGCATGGAGCACGTCA
AGATGTCCTAGATCGCCGTGTGTCCGTCGTCGGTGGATCTCGCAGGGGCGGAGCTGCACC
CACCTGTGATGCTTTCCGTGTCCTTGTGTGTATGTACAGCAGGCCACAGATCCATGTGGT
GAAAACATAAATAACGCGATATGATATGCC

>Clo1-B [organism=Triticum aestivum] EF-hand calcium binding protein,
caleosin mRNA, full CDS
CGGAGCAGCACTCCACAGCTTGCCTGCAAGTTACCTTCTCCACGATTCCGCTCACGGT
TCATCGGTTGCCACCCTGTTCTGGTGCGCCGTCGCAGAGCGGCGAAGATGGCCACCAAGG
GGCGCAAGGTCGAGGTCCGTGACGCGAGCAGGACGGACGGGAAGGGCGACGCCGCGGACG
GGCACGTCGTCCGTGAGGCGAAGGGCGACCACGACACCGCCGGCGGCGTCGGCCACCGGG
GTGCAAACAGGACGGATGGGCATGGCGACGCGGGCAGCAAGATCGAGGACCTCCGGGGCA
TCGGGGGGAAGGACTCCCTGAAGATCGTCGCGATGCAGGCGCCGGTCACCGTGGAGCGCC
CCGTCCGCGGCGACCTCGAGGAGCACGTCCCCAAGCCCTATCTGGCGAGGGCTCTGGCGG
CGCCGGACATGTACCACCCGGAGGGGACGACGGATGATCACCAGCACCACAACATGAGCG
```

TGCTGCAGCAGCACGTCGCCTTCTTCGACCGCGACAACAACGGCATCATCTATCCGTGGG
AGACTTACGAGGGATGCCGTGCGGTTGGGTTCAACGTGTTCATGTCTGCCTTCATCGCGT
TTCTGGTGAACCTGGTCATGAGCTATCCCACCCTGCCCGGTTGGCTGCCCAACCCTCTCT
TCCCTATCTACGTGCACAACATCCACAAGAGCAAGCACGGGAGTGACTCGGGCACCTACG
ACAAAGAGGGCCGGTTCATGCCGGTGAATTTCGAGAACATCTTCAGCAAGTACGCCCGCA
CGTACCCGGACAGGCTGAGCTACAGGGAGCTGTGGCGGATGACCGAGGGGTCCCGGGAGG
TGTTCGATTTCTTCGGCTGGGTGGCGATGAAGCTGGAGTGGTCGATCCTGTACGTGCTGG
CGCGGGACGACGAAGGGTACCTGTCGAGGGAGGCCATCCGGCGCATGTACGACGGCAGCC
TCTTCGAGTACATGGAGAGGCAGCGCATGGAGCACGTCAAGATGTCTTAGATCACAGGCT
CATCGCCGACGGTGGATCTCGCAGGGTCGGAGCTGCACCCGCCTGTGATGCTTTCCGTGT
CCGTGTGTATATGATGTACAGCAGGCCACAGATCCATGTGGTGTACCGCTGGTGTGTAAA
ACATAAATAAGCGATA

>Clo1-D [organism=Triticum aestivum] EF-hand calcium binding protein, caleosin mRNA, full CDS
GGCATCACTCCACAGCTTGCCTGCAAGCCATCTTCTCCACGATTCCGCTCACGGTTCATC
GGTTGCCACCCGATTCTGGTGCGCCGTCGCAGAGCGGCGAAGATGGCCACCAAGGGGCGC
AAGGTCGTCGAGGTCCGTGACGCGAGCAGGACGGACGGGAAGGGCGACGCCGCGGACGTC
CACGTCGTCCGCGAGGGGAAGAGCGACCACGACACCGCCGGCGGCGTCGGCCACCGCGGC
GCGAGCAGGACGGACGGGCATGGCGACGCGGGCGGCAAGGTCGGGGACTTCCGCGGCATC
GACGGGAAGGACTCCCTGAAGATCGTCGCGATGCAGGCGCCGGTCACCGTGGAGCGCCCC
GTCCGCGGCGACCTCGAGGAGCACGTCCCCAAGCCCTATTTGGCGAGGGCTTTGGCGGCT
CCGGACATGTACCACCCGGAGGGGACGACGGATGATCACCAGCACCACAACATGAGCGTG
CTGCAGCAGCACGTCGCCTTCTTCGACCGCGACAACAACGGCATCATCTATCCCTGGGAG
ACTTACGATGGATGCCGTGCGGTTGGGTTCAACGTGTTCATGTCCGCCTTCATCGCGTTT
CTGGTGAACCTGGTCATGAGCTATCCCACCCTGCCCGGTTGGCTGCCGAACCCTCTCTTC
CCGATCTACGTGCACAACATCCACAAGAGCAAGCACGGGAGTGATTCGGGGACCTATGAC
AAAGAGGGCCGGTTCATGCCGGTGAATTTCGAGAACATCTTCAGCAAGTACGCCCGCACG
TACCCGGACAGGCTCAGCTACAGGGAGCTGTGGCGGATGACCGAGGGGTGCCGGGAGGTG
TTCGATTTCTTCGGCTGGGTGGCGATGAAGCTGGAGTGGTCGTTCCTGTACGTGCTGGCT
CGGGACGACGAAGGGTACCTGTCGAGGGAGGCCATCCGGCGCATGTACGACGGCAGCCTC
TTCGAGTACATGGAGAGGCAGCGCATGGAGCACGTCAAGATGTCCTAGATCACAGTCGAC
GGTGAAACTCGCATGGTCGGAGCTGCACCCACCTGTGATGCTTTCCGTGTCCGTGTGTGT
GATGTACAGCAGGCCACAGATCCATGCGGTGTGCCGCTGGTGTGGCGAAATGAAAACATA
AATAACGTGATATATGCCATGGTTTGTATGTTTAGCCC

>Clo2-A [organism=Triticum aestivum] EF-hand calcium binding protein, caleosin mRNA, full CDS
TACGCACAGTGTGATACGCAGAGATAGCGATCGAGCCGAGCGCCATGGCCGAGGAGGCAA
CAAGGGCGGTCAGGGAAGAAGAGCTGTCGCCGGTGGCGGAGGGGGCGCCCGTGACGGCCC
GGCGGCCCGTCCGAGCGGACCTGGAGAAGCGCATCCCGAAGCCCTACCTGGCCCGAGCCC
TGGTGGCGCCGGACGTGTACCATCCCGGAGGGAGCAAGGAGGGCGGGCACCAGCACCGCC
AGAGGAGCGTGCTGCAGCAGCACGTCGCCTTCTTCGACATGGATGGCGACGGCGTCATCT
ATCCATGGGAAACTTACCAAGGACTGAGGGCATTGGGCTTCAACATGATCGTCTCCATCC
TCATCGCAATAGGCATACATACTACCCTGAGCTACACAACTCTGCATAGCTGGGTGCCAT
CTCTCCTTTTCCCAATCTACATCGACAACATCCACAGGGCCAAGCATGGGAGCGACACCG
CGACTTATGACTCCGAGGGAAGGTACATGCCGGTGAACTTCGAGAACATATTCAGCAAGA
ACGCCTCGCCGGATAAGCTCACATTCCGGGAAATCTGGATGATGACCGACGGCCAACGGC
AGGCGAACGACCCATTTGGATGGGTGGCGAGCAAGGGGGAGTGGATGCTGCTGTACATGC

TCGCCAAGGACGAGGAGGGGAACCTTCCGAGGGAGGCTATTCGCCGCTGCTTCGACGGTA
GCCTCTTCGAGTTCATCGCCGACGAGAGGAGGCAGGCACACGGGAAGCGGCAGTAGCATG
CAACGCCTGCCGGCCCGGAACGTCTTCATGTGTATTGTGTAAGACTGTGGACTAGCTACC
GCCTCATGTATTCCTACCATGAAGTAATAAGTTGTGCCCTCCTCAAAGTTTCATCAGGAA
AAGAAACACCGTCGTCGTTTTCAATGATAATTTGAAACAATTGAGGCTGCTCGCATGGAA
TGC

>Clo2-B [organism=Triticum aestivum] EF-hand calcium binding protein, caleosin mRNA, full CDS
CATTGATCCCTCCGCAACGCACGGTGTGATACAGAGAGAGCGATCGAGCCGAGCGCCATG
GCGGAGGAGGCTAGGGCGGTCAGGGAAGAAGAGCTGTCGTCGGTGGCCGAGGGGGCTCCC
GTGACGGCCCGGCGGCCCGTCCGAGCAGACCTGGAGAAGCACATCCCAAAGCCCTACCTG
GCCCGAGCTCTGGTGGCGCCGGACGTGTACCATCCTGGAGGGAGCAAGGAGGGCGGTCAC
CAGCACCAGCAGAGGAGCGTGCTGCAGCAGCATGTCGCCTTCTTCGACATGGATGGCGAC
GGCGTCATCTATCCATGGGAAACTTACCGAGGACTGAGGGCACTGGGCTTCAACATGATC
GTGTCCGTCCTCATCGCAATAGGCATACATACTACTCTGAGCTACACAACTCTGCCTAGC
TGGGTACCATCTCTGCTGTTCCCAATCTACATCGACAACATCCACAGGGCCAAACATGGC
AGCGACACCGCGACTTATGACTCCGAGGGAAGGTACATGCCGGTGAACTTCGAGAACATA
TTCAGCAAGAACGCCCGCTCGTCGCCGGATAAGCTAACGTTCCGGGAGATCTGGATGATG
ACCGACGGCCAACGGCAGGCGAACGACCCATTTGGATGGGTGGCGAGCAAGGGGGAGTGG
ATGCTGCTGTACATGCTCGCCAAGGACGAGGAGGGGAACCTTCCGAGGGAGGCCATTCGC
CGCTGCTTCGACGGTAGCCTGTTCGAGTTCATCGCCGACGAGAGGAGGCAGCCACACGGG
AAGCAGCAGTAGCATGCAACGCTGCCGGCCCGGAATGTCTTCCTGTCTAAGACTGTGTGT
GGACTGTGGACTAGCTACCACATCCTCATGTACTCCTACCATGAAGTAATAAGTTGTGCC
CTCCTCAAAGTTTCATCAGGAAAAGAAACACAGTCGTTTTCAATGATAATTTTAAACAAA
AAAAAAAAAAAAAAAAAAAAAAAAAA

>Clo2-D [organism=Triticum aestivum] EF-hand calcium binding protein, caleosin mRNA, full CDS
TCCGCAACGCACGGTGTGATACAGAGAGAGCGATCGAGCCGAGCGTCATGGCGGAGGAGG
CAAGGGCGGTCAGGGAAGAAGAGCTGTCGCCGGTGGCGGAGGCGGCGCCCGTGACGGCCC
GGCGACCCGTCCGAGCGGACCTGGAGAAGCACATCCCGAAGCCCTACCTGGCCCGAGCCC
TGGTGGCGCCGGACGTGTACCATCCCGGAGGGAGCAAGGAGGGCGGGCACCAGCACCGCC
AGAGGAGCGTGCTGCAGCAACACGTCGCCTTCTTCGACATGGATGGCGACGGCGTCATCT
ATCCATGGGAAACTTACCAAGGACTGAGGGCACTGGGCTTCAACATGATCGTCTCCGTCG
TCTTCGCAATGGCCATTCATGCTAGCCTCAGCTACACAACTCTGCATAGCTGGGTACCAT
CTCTGCTGTTCCCGATCTACATCGACAACATCCACAGGGCCAAGCATGGCAGCGACACCG
CGACTTATGACACCGAGGGAAGGTACATACCGGTGAACTTCGAGAACATATTCAGCAAGA
ACGCCCGCTCGTCGCCGGATAAGCTCACATTCCGGGAGATCTGGATGATGGCCGAGGGCC
AACGGCAGGCGAACGATCCATTCGGATGGGTGGCGAGCAAGGCGGAGTGGATGCTGCTGT
ACATGCTCGCCAAGGATGAGGAGGGGAACCTTCCGAGGGAGGCTATTCGCCGCTGCTTCG
ACGGTAGTCTCTTCGAGTTCATCGCCGACGAGAGGAGGCAGGCACACGGGAAGCGGCAGT
AGCATGCAACGCTGTCGGCCCGGAATGTCTTCCTGTCTAAGACTGTGGACTAGCTACCGC
CTCATGTATTCCTACCATGAAATAATAAGTTGTGCCC

>Clo3-A [organism=Triticum aestivum] EF-hand calcium binding protein, caleosin mRNA, full CDS

GGCGGCGGCAGCAGCAGCAGCGATGGCGATCCGGCGACAATCATCAGCAGCAGCTTCTCC
ACTCCTGCCGCCGGTGGCTGCTCTTCTGTTCCTATGGATGTTTGGCGGGGGGCGTGTGAT
GGCACATACTGAGATTCGCAACATGACGGCACTCCAGAAACATGTCTCCTTTTTCGACCG
TAACAAGGATGGCATCATTACCCCTTCGGAAACATTTGAAGGGTCTGTCGCAATTGGTTT
TAATGTTACATATGCCAGAGAATTTGCCACCTTGGTGCATGCTGCTAATGGTCCTATAAC
AAGCCCCGCTGATGCACCATTGCCTCACTTATCAATATACATAGAGAATATGCACAAAGG
AATGCATGGGAGTGATACCGGTGCATTTGATGTTAAAGGAAGGTTTGTTCCACAAAAGTT
TGAGGAAATATTCGTAAAGCATGCAAAAACTAGACCAGATGGTTTGACATATTTGGAGTT
GGAGGATATGATCCTAGCAAATCGAGATCCACTGGACCCTGCATCATGGGAGGGACCTCA
AATAGAATGGGGCGGAATATACAACGTCACGAGTGACAATGATGGATTTCTTCATAAGGA
CGATGCGAGAGGTATATACGACGGAAGTGTGTTTGTAAAGCTGGAGGAAAAGAGGGCCTT
TTCTCATCATAGTGCAATGTAATAGAGTGCAACATGTTGTACGCTGAAATAATT

>Clo3-B [organism=Triticum aestivum] EF-hand calcium binding protein, caleosin mRNA, full CDS
TCTGCTTCAGCAAGAAAGAGGGACCTTTCATATGTAGCTCTTCCCTGGCTACTTAAACAG
TGCACCGCGTTCCCTGAGCACACTCTCCAGTCACACGGCCAGAGAAGAAGGGGTTACCGG
CGGCAGCAGCAGCAGCGATGGCGATCCGGCGACAATCATCATCAGCAGCTTCTCTACTCC
TGCCGCCGGTGGCTGCTCTTCTGTTTCTATGGATGTTTGGCGGGGGGCATGTGATGGCAC
ATACTGAGATTCGCAACATGACGGCTCTCCAGAAACATGTCTCCTTTTTCGACCGTAACA
AAGATGGCATCATCACTCCTTCGGAAACATTTGAAGGGTCTGTCGCAATTGGTTTTAATG
TTACATATGCGAGAGAATTTGCCACCTTGGTGCATGCTGCTAATGGTCCTATAACAAGCC
CCGTTGATGCACCATTGCCTCACTTATCAATATACATAGAGAATATGCACAGAGGAATGC
ATGGGAGTGATACCGGTGCATTTGATGTTAAAGGAAGGTTTGTTCCACAAAAGTTTGAGG
AAATATTCATAAAGCATGCAAAAACTAGACCAGATGGTTTGACATATTTGGAGATGGAGG
ATATGATCCTAGCAAATCGAGATCCACTGGACCCTGCATCATGGGAGGGACCTCAAATAG
AATGGGGCGGAATATACAACGTCGCGAGTGACAATGATGGATTTCTTCATAAGGACGATG
CGAGAGGTATATACGACGGAAGTGTGTTTGTAAAGCTGGAGGAAAAGAGGGCCTTTTTCT
CATCATAGTGCAATGTAATAGAGTGCAACCATGTTGTACGCTGAAATAATTAGGGGAACA
CATAGTGTGTGTATCTAAGACTGGTATATATTTGTTCAAGTGTTTGTTTGCACATAAGTA
AAATACT

>Clo3-D [organism=Triticum aestivum] EF-hand calcium binding protein, caleosin mRNA, full CDS
CGGACAGAGAAGAAGGGGTTACCGGCGGCGGCAGCAGCAGCGATGGCGATCCGGCGACAATCATCAGCAG
CAGCTTCTCTACTCCTGCCGCCGGTGGCTGCTCTTCTGTTTCTATGGATGTTTGGCGGGGGGCATGTGAT
GGCACATACTGAGATTCGCAACATGACGGCACTCCAGAAACATGTCTCCTTTTTCGACCGTAACAAGGAT
GGCATCATTACTCCTTCGGAAACATTTGAAGGGTCTGTCGCAATTGGTTTTAATGTTACATATGCGAGAG
AATTTGCCACCTTGGTGCATGCTGCTAATGGTCCTATAACAAGCCCCGCTGATGCACCATTGCCTCACTT
ATCAATATACATAGAGAATATGCAGAGAGGAATGCATGGGAGTGATACCGGTGCATTTGATGTTAAAGGA
AGGTTTGTTCCACAAAAGTTTGAGGAAATATTCATAAAGCATGCAAAAACTAGACCAGATGGTTTGACAT
ATTTGGAGGTGGAGGATATGATCCTAGCAAATCGAGATCCACTGGACCCTGCATCATGGGAGGGACCTCA
AATAGAATGGGGCGGAATATACAACGTCGCGAGTGACAATGATGGATTTCTTCATAAGGACGATGCGAGA
GGTATATACGATGGAAGTGTGTTTGTAAAGCTGGAGGAAAAGAGGGCCTTTTCTCATCATAGTGCAATGT
AATAGAGTGCAACATGTTGTGGGCTGAAATAATTAGGGGAACACATAGTGTGTGTACTAAGACTGGTATA
TATTTGTTCAAGTGTTTGTTTGCACATAAGTAAAATACTGTGATGTTTATCAAGATATATATATGAACTC
TGAAAAAAAAAAAAAAAAA

>Clo4-A [organism=Triticum aestivum] EF-hand calcium binding protein, caleosin mRNA, full CDS
CACGATGGCCGGTCATCGACAATCGTTGGCAGCGGCTTCTCTGAAGCTCCCAGCTCTTCT
GCTTCTGTGGATCTTTAGCTTGAACTGGGGGCATGCCGTCGCGCACTTTGATCCTGCAAA
CATGACGGAACTGCAGAAACATGTCTCCTTTTTCGACCGCAACAAGGATGGCTTCATCAC
TCCTGTGGAAACCATCCAAGGGTTTGTTGCAATCGGTTGCGAGTATGCATTTGCTACTGC
TGCCTCAGCCTCCATTCATGGTGCCCTTGCTCCTCAAACAACCCCGGCTGGCACACCACT
GCCTCACTTGACAATATACGTGGAGAATATCCACAAAGCTATGCATGGAAGTGATTCGGG
TGTATACGATCCTAAAGGAAGGTTTCTTCCCCAAAAGTTTGAGGAATTATTCAAAACATA
TGCAATACTCCGGCCAGATGCGTTGACACTTGCAGAGATGCATGCGATGCTCTTTGCTAA
ACGAGATCTAGACCCGATATCATGGGCGCCACCCGAGATAGAGTGGGGGCTATTATTCAC
GCTTGCAAGCGATTGGCTTGGATTTCTTCACAAGGACAGTGTTAGAGGTATATACGATGG
AAGCGTGTTTACCAAGTTGGAGAAGAAATGGCACCCTTCTCAAAGTGATATATGATGAAC
TTGATGTAATGTGGGACCCACATACAAGCGACGGAGAAGGCCCAATTCTTCCACACTAGG
AGACCATGTCACTGCAGGCAACCACCCAATTTGAAATACATATAGTATCTCTAATTACCA
TAGTTAATACCCACCCTCCGTCTTAATATAAGTGTCTCAACTCTAATACTACTTTATAGT
AAAGTTAGTACAAAGTTGAGACATTTATTTTGGGATGGAGGAAGTATTAGTTTAGAGTAA
GAGTTTGGATATGGAAAGGTTCGTCCCGAAGAAGGTTTTTCTACTATCTCCAAATTCAAC
TAGAGTTTTTTCTTTTCCTCCAAGTTGTAATTTGCTTTATAAGCCCCCCAGAGTCGATCA
ATATGATCAGGCAAGTGAA

>Clo4-B [organism=Triticum aestivum] EF-hand calcium binding protein, caleosin mRNA, full CDS
TCGTTGAGGCGACGATGGCCGGTCATCGACAATCGTTGGCAGCGGCTTCTCTGAAGCTCCCAGCTCTTCTGCTTCTG
TGGATCTTCAGCTTGAACTGGGGGCATGCCGTCGCGCACTTTGATCCTGCAAACATGACCGAACTGCAGAAACATGT
CTCCTTTTTCGACCACAACAAGGATGGCTTCATCACTCCTGTGGAAACCATCCAAGGAGGGTTTGTTGCAATCGGTT
GCGAGTATGCATTTGCTACTGCTGCCTCAGCCTCCATTCACGGTGCCCTTGCTCCTCAAACAACCCCGGCTGGTACA
CCACTGCCTCACTTGACAATATACGTGGAGAATATCCACAAAGCTATGCATGGAAGTGATTCGGGTGTATACGATCC
TAAAGGAAGGTTTCTTCCCCAAAAGTTTGAGGAATTATTCAAAACATATGCAATACTCCGACCAGATGCGTTGACAC
TTGCAGAGATGCATGCGATGCTCTTTGCTAAACGAGATCTAGACCCGATATCATGGGCGCCACCCGAGATAGAGTGG
GGGCTATTATTCACGCTTGCAAGCGATTGGCTTGGATTTCTTCACAAGGACAGTGTTAGAGGTATATACGATGGAAG
CGTGTTTATCAAGTTGGAGAAGAAATGGCACCCTTCTCAAAGTGATATATGATGAACTTCACGTAATGTGGGACCCA
CATACAAGCGACGGAGATGGCCCAATTCGTCCACACCAGGAGTCCATGTCATTGCAGACAACCACGCAATTTGAAAT
ACATATTATATCTCTAGTTACCATACTTAATACCCCCCTCCATCTCAAAATAAGTGTCTCAACTCTAATACTACTTT
ATATTAAAGTTAGTACAAAGTTGAGACATTTATTTTGGGATGG

>Clo4-D [organism=Triticum aestivum] EF-hand calcium binding protein, caleosin mRNA, full CDS; Derived from WSS
TCGTTGCAGGCGACGATGGCCGGTCATCGACAATCGCTGGCAGCGGCTTCTCTG
AAGCTCCCAGCTCTTCTGCTTCTGTTGATCTTCAGCTTGAACTGGGGGCATGCCGTCGCG
CACTTCGATCCTGCAAACATGACGGAACTGCAGAAACATGTCTCCTTTTTCGACCGCAAC
AAGGATGGCTTCATCACTCCTGTGGAAACCATCCAAGGGTTTGTTGCAATCGGTTGCGAG
TATGCATTTGCTACTGCTGCCTCAGCCTCCATTCATGGTGCCCTTGCTCCTCAAACAACC
CCGGCTGGTACACCACTGCCTCACTTGACAATATACGTGGAGAATATCCACAAAGCTATG
CATGGAAGTGATTCGGGTGTATACGATCCTAAAGGAAGGTTTCTTCCCCAAAAGTTTGAG
GAATTATTCAAAACATATGCAATACTCCGACCAGATGCGTTGACACTTGCAGAGATGCAT
GCGATGCTCTTTGCTAAACGAGATCTAGACCCGATATCATGGGCGCCACCCGAGATAGAG
TGGGGGCTATTATTCACGCTTGCAAGCGATTGGCTTGGATTTCTTCACAAGGACAGTGTT
AGAGGTATATACGATGGAAGCGTGTTTACCAAGTTGGAGAAGAAATGGCACCCTTCTCAA
AGTGATATATGATGAACTTGATGTAATGTGGGACCCACATACAAGCGACGGAGAAGGCCC
AATTCGTCCACACCAGGAGTCCATGCCACTGCAAGCAACCACCAAATTTGAAATACGTAT

TGTATCTCTAGTTACCGTATTTAATACCCCCCTCCATCTCAAAATAAGTGTCTTAACTCT
AATACTACTTTATATTAAAGTTAGTACAAAGTTGAGACATTTATTTTGGGATGGAGGGAG
TATTAGTTTAGAGTAAGGGTTTGGATATGGAAAGGTTCGTCCCGAAGAAGGTTTTCCTAC
TATCTCCAAATTCAACTAGAGTTTTTTCTTTTCCTCCAAGTTGTAATTTGCTTTATAAGC
CCTCCAGAGTCGATCAATAAGATCACGCAAGGTGAAACTTTTTCCTACCGTGTCTANAAA
AAAAAAAAAA

>Clo5-A [organism=Triticum aestivum] EF-hand calcium binding protein, caleosin mRNA, full CDS
AGCGAGCGAGGCGGAAGAATCCCTCGAGATGGACGCAAGACCGCGACGGGCGTCGTCCTC
CCCGGCGGCGGCGACGGCGACGGCGGCGGCGGCCTTGTCCCTGCTGCTCCTGTTCCCCAT
GTTCCTAGGGGGCCAGGCGTCGGGGTACGGCGACGATTCGGGCGCCGGCGGCATGACGGC
GCTGCAGAAGCACGCGGCCTTCTTCGACCGCGACAAGGACGGCGTCGTCACCTTCTCCGA
GACGTACGCAGCGTTTCGGGCCCTCGGATTTGGATTTGCTTCGTCCACCTTGAGTGCCAC
CTTCATCAATGGCGTCCTTGGCCCCCAGACCAGACCGGAAAATGACACGGCGCGCATGTC
CATCTACATCGAGAACATCCACAAGGGCATCCACGGGAGCGACTCAGGCGCGTATGACTC
CCAAGGAAGGTTCGTTCCGGACAAGTTCGATGCGGCGTTCGCCAAGCACGGCAAGACGGT
GCCGGACGCCCTGACGTCCGCGGAGGTGGACGAGCTGATCGCCGCGAACCGGCAGCCCGG
CGACTACGCGGGATGGGCGGGCGCGTCGGCGGAGTGGAAGCTGCTGTACAGCATCGGCAA
GGACGAGGACGGGCTCCTCCGCAAGGACGCCGCCAGGGGCGTCTACGACGGCAGCCTCTT
CGCCAGGGTGGTGCAGGAGCGGAGGGCATCCTCGCTAGAAGAAACCCAGGCATGATCGTG
GCACGCCGTCACGGTCTGTGTGTGTGTGTGGCTACCGTACAAGCGACGGAGGCCAGTCTG
AACACTACTCGAGTTCGTCCGAACTGATGCGTCGCTGGCTAGGTACAGGGCATTACTGGT
ACTCCCCCGTCACGGTTAGAAGGCGTCTTCGAAATTCTCTTCGGACTAGGCGGATATCTA
TGATTATAGATGGGTTAAAAATAGCATCACGCTACCATACATTTGAATCGTACATCGGAT
TCTATTAGCTACAGAATTCATGTATAAATCCAAACCTGGTTTTGGGAAAACCCCGTAATT
AACTGTGGCTTAAATTTTGACGGGGGGTATATA

>Clo5-B [organism=Triticum aestivum] EF-hand calcium binding protein, caleosin mRNA, full CDS
CGCGGCAAAGACACAGAGAAGAGAGGTGACAACAGTCAAAAGCGAGCGAGGCGGAAGAAA
TCCCTTGAGATGGATGCAAGACCGCGACGGGCATCGTCCTCGCCGGCGGCGGCGACGGCG
GCCTTGTCCCTGCTGCTCCTGTTCCCCATGTTCTTAGGGGGGCAGGCGTCGGCGTACGGC
GACGATTCGGGCGCCGGCAGCATGACGGCGCTGCAGAAGCACGCGGCGTTCTTCGACGGC
GACAAGGACGGCGTCGTCACCTTCTCCGAGACATACGCAGCGTTTCGGGCCCTTGGATTC
GGGTATGCCGCCTCCACCTTGAGTGCTACCTTCATCAATGGCGTCCTTGGCCCCCAGACC
AGACCGGAAAATGATACGGCGCGCATGTCCATCTACATCGAGAACATCCACAAGGGCATC
CATGGGAGCGATTCAGGCGCGTATGACTCTCAAGGAAGGTTCGTTCCCGAGAAGTTCGAG
GCGGCATTCGCCAAGCACGCCAAGACGGTGCCGGACGCCCTGACGTCCGCGGAGGTGGAC
GAGCTGATCACCGCGAACCGGGAGCCCAGCGACTACGCGGGATGGGCGGGCGCGTCGGCG
GAGTGGAAGTTGCTGTACAGCATCGGCAAGGACAAGGACGGGCTCCTCCGCAAGGACGCC
GCCAGGGGCGTCTACGACGGCAGCCTCTTCGCCAGGGTGGTGCAGGAGCGGAGGACATCT
ATAGGAGAAACCCAGGCATGATCGTGGCACGCCGTCACGGTGTGTGGCTACCGTACAAGC
GACGGAGGCCAGTCGAACACTCGAGTCCGTCCGAACCGATGCGTCGCTGGCTAGTTACAG
GGCATTACTTATCCGGTGTGATTGGCTCGTCGCGTCACGCCGTCACGGTCTGTCGCCACA
TAGCTACGGACGGTACTATACTGCTGAATTAATGTTTAAAAAAAATAGGCAGTGTGAAAGG
GTGTGAACGCTTGTGAACGGAATCTTGGGATGGGACCGATGTCCTGTTGTTTGTGTCCTG
CGGATATGGTTTTATTATGCCCACACGACTG

>Clo5-D [organism=Triticum aestivum] EF-hand calcium binding protein, caleosin mRNA, full CDS
GAAGCGAGAGGGGACAACAGTCAAAAGAGCGAGCGAGTCGGAAGAAATCCCGAGATGGAC

GCAAGACCACGACGGGCATCGTCCTCGCCGGCGGCGGCCTTGTCCCTGCTGCTCCTGTTC
CCCATGTTTCTAGGGTGCCAGGCGTCGGCGTACGGCGACGATTCGGGCGCCGGCGGCATG
ACGGCGCTGCAGAAGCACGCGGCGTTCTTCGACGGTGACAAGGACGGCGTCGTCACCTTC
TCTGAGACTTACGCAGCGTTTCGGGCCCTCGGATTTGGATTTGCTGCCTCCACCTTGAGT
GCCACCTTCATCAATGGCGTCCTTGGCCCCCAGACCAGACCGGAAAATGACACGGCGCGC
ATGTCCATCTACATCGAGAACATCCACAAGGGCATCCACGGGAGCGACTCAGGCGCGTAT
GACTCCCAAGGAAGGTTCGTTCCGGACAAGTTGGAGGCGGCGTTCGCCAAGCACGGCAAG
ACGGTGCCGGACGCCCTGACGTCCGCCGAGGTGGACGAGCTGATCACCGCGAACCGGCAG
CCCAGCGACTACGCGGGATGGGCGGGCGCGTCGGCGGAGTGGAAGCTGCTGTACAGCATC
GGCAAGGACAAGGACGGGCTCCTCCGCAAGGAGGACGCCAGGGGCGTCTACGACGGCAGC
CTCTTCGCCAGGGTGGTGCAGGAGCGGAGGGCATCATCGCAAGAAGAAACCCAGGCATGA
TCGTGGCACGCCGTCACGGTGTGTGTGTGGCTACCGTACAAGCGACGGAGGCCAGTCTGA
ACACTCGAGTCCGTCCGAACCGATGCGTCGCTGGCTAGGTACAGGGCATTACTGGTACTA
GTCCAAATTACTTATCCGGTGTGATTGGCTCGTCGCGTCACGCCGTCACGGTCTGTCGCC
ACATAGCTACGGACAGTACTATACTGCTGAACGAATGTTAGAAAAATAGCCAGTGTGAAC
AGGTGTGAACAGGTGTGAACGCTTGTGAACGGAATCTTTGGGATGGGACCGATGTCCTCT
TGTTTGTGTCCTGTGGATATGGATTTACAATGCGTGCACGACTGACTTGCTAGGCGGTGC
AGTTGTATGGGCTATGGAGTACTCCTACTCCTTTGTTTCGTTGTAAAAAAAAAAAAAAAA
AAAAAAAAAA

>Clo6-A [organism=Triticum aestivum] EF-hand calcium binding protein, caleosin mRNA, full CDS
GATAGATCGGGAGTCAGCTTGGGAGAGAGAGAGAGAGGGTCGAGAGAGAGGAGGAATCGC
CAGACACGATGGGCGCCACCGGCCAACGTCGGCTGTCATCTCTGCCCGCGGCGGCGGCCG
CGCCCCTCCTGCTTCTGCTCGTCGTGTCCTCCTGGAGCCAGGCGGCGGCGGCGGCTCCG
GTCTGGACGACGGCGCTGGAGAAGCACGTGGCGTTCTTCGACACCGACAACGACGGCATC
GTCTCCTTCTCCGAGACCGAGCAAGGGCTTCGTGCCATCGGTCTCGGAGCTATCGAGGCG
GCGGCCAGCGCGACCCTGATCAACGGAGCCATCGGACCCAAGACCAGACCTGAAAATGCT
ACGACGTCGCGGTTTGACATCTACATAGCCAACATCCATAAAGGGATCCACGGGAGCGAC
AGCGGCTCGTACGATGCTCAAGGAAGGTTCGTTCCGGCCAAGTTCAACGACATATTCGCC
AAGTACGCCAAGGTCAAGCCGAACGCCCTGAACGAGGACGAGCTGGGGGAGATGCGCACT
GCCAACAGGAAGGAGGGTGACTTCAAAGGATGGGCGGCGTCGAAGGCGGAGTGGGGCATG
CTCTACAGCCTCGCCAAGGACAAGGACGGCTTCCTTCAGAAGGACACCGCGCGCAGCGTC
TACGACGGCAGCCTCTTCGCTAAGCTGGCCAAGAAGGCTGCTTCGTCTGGAAATTAACCG
ACCCCGACCGTGATTTGTACCCCGTGTTTTTCTTCGAGATGGAATTTGTACTCCGTATT
ATTCGTGTCTTGTTCGTTGAACCATGTGCTGTCGCTGTACAATTAAAA

>Clo6-B [organism=Triticum aestivum] EF-hand calcium binding protein, caleosin mRNA, full CDS
GTCGAGAGAGAGCGAGGAGGAATCGCCTGCCCTAACACGATGGGCGCCACCGGCCAGCGT
CGGATGTCATCTCTGCCCGCGGCGGCGGCCGCGCCTCTCCTGCTTCTGCTCGCCGTGTCC
TCCTGGAGCCAGGCGGCGGCGGCTCCGGCCTGGACGACGGACCTGGAGAAGCACGTGGCG
TTCTTCGACACCGACAACGACGGCATCGTCACCTTCTCCGAAACCGAGCAAGGGCTTCGT
GCCATCGGTCTCGGAGTTCTCGAGGCGACGGCCAGCGCGACCCTGATCAACGGAGCCATC
GGACCCAAGACCAGACCTGAAAATGCTACGACATCGCGGTTTGACATCTACATAGCGAAC
ATCCAGAAGGGGATCCACGGGAGCGACAGCGGTTCGTACGATGCTCAAGGAAGGTTTGTT
CCGGCCAAGTTCAACGAGATATTCACCAAGTACGCCATGGTCAAGCCGAACGCCCTGAAC
GAGGACGAGCTGGATGCGATGCGCACTGCCAACAGGAAGGAGGGTGACTTCAAAGGATG
GGCGGCGTCGAAGGCGGAGTGGGGCATGCTCTACAGCCTCGCCAAGGACAAGGACGGCT

TCCTTCAGAAGGACACCGTGCGCACCGTCTATGATGGTAGCCTCTTCGCTAAGCTGGCGA
AGAAGGCTGGAAATTAACCGAGCGTGATTTGTATTCGTACTTCTTTTCGAGATGAAATTT
GTACCCCGTGTTATTGGGGTCTTGTTTGTTGAGCATGAGCTGTCCCTGTACAATTAAAAT
TGAAATCTGTTAGTGCAAATATAATTTACTGTTCCATTAAAAAAAAAAAAAAAAAAA

>Clo6-D [organism=Triticum aestivum] EF-hand calcium binding protein, caleosin mRNA, full CDS
AGAGGGAGGAGGAATCGCCTGACACGATGGGCGCCACCGGCCAGCGTCGGATGTCATCTCTGCCCGCGGCGGCGGCC
G
CGCCTCTCCTGCTTCTGCTCGCCGTGTCCTCCTGGAGCCAGGCGGCGGCGGGCGGCAGCG
CTCCGGCCTGGACGACGGCGCTGGAGAAGCACGTGGCGTTCTTCGACACCGACAACGACG
GCATCGTCTCCTTCTCCGAGACCGAGCAAGGGCTTCGTGCCATCGGTCTCGGAGCTATCG
AGGCGGCGGCCAGCGCGACCCTGATCAACGGAGCCATCGGACCCAAGACCAGACCTGAAA
ATGCTACGACGTCGCGGTTTGACATCTACATAGCCAACATCCAGAAAGGGATCCACGGGA
GCGACAGCGGCTCGTACGATGCTCAAGGAAGGTTCGTTCCGGCCAAGTTCAACGACATAT
TCACCAAGTACGCCAAGGTCAAGCCGAACGCCCTGAACGAGGACGAGCTGGATGCGATGC
GAACTGCCAACAGGAAGGAGGGTGACTTCAAAGGATGGGCGGCGTCGAAGGCGGAGTGGG
GCATGCTCTACAGCCTCGCCAAGGACAAGGACGGCTTCCTTCAGAAGGACACCGTGCGCA
CCGTCTACGACGGTAGCCTCTTCGCTAAGCTGGCGAAGAAGGCTGGAAATTAACCGAGCG
TTATTTGTATTCGTACTTCTTTTCGAGATGAAAATTGTACCCCGTATTATTTGGGTCTTG
CTCGTTGAGCATGAGTTGTCCCTGTACAATTAAAA

>Clo7-A [organism=Triticum aestivum] EF-hand calcium binding protein, caleosin mRNA, full CDS
CCACCTTGGGACCAGTGCCTTTCCCTCCTCCCTCTCTCGCAACCTTTCCCCCCGTCTCGT
TGGAATCCATGGCGTCCAAGTCCGCGGTCACCACCGCAGGTGGGCAGCTGCCCAAGGGCA
AGGAGGAGTTCTCCTCCATGGCGGACGTGTACAACCACGAGCTGACGCCGCTGCAGAAGC
ACGTCGCCTTCTTCGACCGCAACAAGGACGGCGTCATCCACCCCTCCGAGACCTACGAAG
GGTTCCGCGCGATCGGGTGCGGCGTCGCGCTGTCCGCCTTCAGCGCAGTCTTCATCAACG
GACTGCTCGGTCCCAAGACCATACCGGAGAACATGAAGGTTGGAGCTTTCAAGTTTCCGA
TCTACGTAAAGAACATCCACAAGGGCAAGCATGGCAGCGATTCGGGCGTGTACGATGCCA
ATGGAAGGTTTGTTCCTGAAAAGTTTGAAGAGATATTCAAAAAGCATGCTCACACCAGGC
CTGATGCCCTGACAGGCAAAGAGCTGAATGAGTTGCTTCAAGCAAACAGGGAGCCTAACG
ACTTGAAAGGACGAGTGGGTGGCTTCACGGAGTGGAAAGTTCTCTACTCGCTGTGCAAAG
ATAAGGAGGGATTTCTTCACAAGGAGACTGTCAGGGCATCTACGATGGCAGCCTGTTTG
TGAAGTTGGAGCAAGAGAGGAAGCAAGCTAAGGAATCTGCCAAGAAGAAATGATGAAAAC
TATCCCAATACCCTGTTATTTGTGATTGTGCGCAAGTATGCGTAAATTATGGTGTGCTTG
CGAGTATGGTTCTGTAAATTAATGTTGTGATTTGATTTGGTGTGTTTG

>Clo7-B [organism=Triticum aestivum] EF-hand calcium binding protein, caleosin mRNA, full CDS
TTCGGCACGAGGCACAAGTCCCCGATCGCAACCGCCCTCTGTCCTCTCCACCTCCTCGCC
ACCACCTTGGCATCAGTGCCTCTCCCTCCACCCTCTCCCTGCAACTTTTCCCCTCATCTC
GTTGGAATCCATGGCGTCCAAGTCCGCGGTCACCCCAGGTGGGCAGCCCAAGGGCAAGGA
GGAGCCCTCCTCCATGGCGGACGTGTACAACCACGAGCTGACGCCGCTGCAGAAGCACGT
CGCCTTCTTCGACCGGAACAAGGACGGCGTCATCCACCCCTCCGAGACCTACGAAGGGTT
CCGCGCGATCGGGTGCGGCGTCGCGCTGTCCGCCTTCAGCGCCGTCTTCATCAACGGACT
GCTCGGTCCCAAGACCATACCGGAGAACATGAAGGTTGGAGCTTTCAAGTTTCCGATCTA

CGTAAAGAACATCCACAAGGGCAAGCATGGGAGCGATTCGGGCGTGTACGATGCCAATGG
AAGGTTTGTTCCTGAAAAGTTTGAAGAGATATTCAAAAAGCATGCTCACACCAGGCCTGA
TGCGCTGACAGGCAAAGAACTGAATGAGTTGCTTCAAGCAAACAGGGAGCCTAACGATTT
GAAAGGACGAGTGGGTGGCTTCACGGAGTGGAAAGTTCTCTACTCGCTGTGCAAAGATAA
GGAGGGATTTCTTCACAAAGAGACCGTCAGGGCAGTCTACGATGGCAGCCTGTTTGTGAA
GTTGGAGCAAGAGAGGAAGCAAGCTAAAGAATATGCCAAGAAGAAATGATGAAAACTATC
CCAATACCCCTCTTATTTGTGATTATGCGCAAGTATGCGTAAATTATGGTGTGCTTGCGA
GTATGGTTCTGTAAATTAATGTTGTGATTTGATTTCGTGTGTTTGCTTGTTCTTTTATGG
AATGAGAATTCAAAGCC

>Clo7-D [organism=Triticum aestivum] EF-hand calcium binding protein,
caleosin mRNA, full CDS
ATCGCATTCGCAACCGCTCTCCGTTTATATCCCCGGCCTCACCACCTTGGCATCACTACTTCTCCCTCCTCGCAACC
TTTCCCCCCGTCTCGTTGGAATCCATGGCGTCCAAGTCCGCGGTCACCGCAGGTGGGCAGCCCAAGGAGAAGGAGGA
GCCGTCCTCCATGGCGGACGTGTACAACCACGAGCTGACGCCGCTGCAGAAGCACGTCGCCTTCTTCGACCGGAACA
AGGACGGCGTCATCCACCCCTCCGAGACCTACGAGGGGTTCCGCGCGATCGGGTGCGGCGTCGCGCTGTCCGCCTTC
AGCGCCGTCTTCATCAACGGACTGCTCGGTCCCAAGACCATACCGGAGAACATGAAGGTTGGTGCTTTCAAGTTTCC
GATCTACGTAAAGAACATCCACAAGGGCAAGCATGGGAGCGATTCGGGCGTGTACGATGCCAATGGAAGGTTTGTTC
CTGAAAAGTTTGAAGAGATATTCAAAAAGCATGCTCACACCAGGCCTGATGCCCTGACAGGCAAAGAACTGAATGAG
TTGCTTCAAGCAAACAGGGAGCCGAACGATTTGAAAGGACGAGTGGGTGGCTTCACAGAGTGGAAGGTTCTCTACTC
GCTGTGCAAAGATAAGGAGGGATTTCTTCACAAGGAGACCGTCAGGGCGGTCTACGATGGCAGCCTGTTTGTGAAGT
TGGAGCAAGAGAGGAAGCAAGCTAAAGAATCTGCCAAGAAGAAATGATGAAAACTATCCCAATACCCTCTTATTTGT
GATTATGCGCAAGTATGCGTAAATTATGGTGTGCTTGCGAGTATGGTTCTGTAAATTAATGTTGTGATTTGATTTCG
TGTGTTTGCTTGTTCTTTTATGGAATGAGAATTCAAAGCCTGTTTGTCTAGATATCGCATTTGTTTACCAAATATGA
TGTTTTCAGTTGACATTTTCGATTACTAAAAAGT

>Clo8-A [organism=Triticum aestivum] EF-hand calcium binding protein,
caleosin mRNA , full CDS
CCACGCGTCCGGGAAACAGCGTGCATGGCTAGCTGGCTGGTGCCCGCGGCGCACGGCACC
CATGGATGGAGCACGTACTACATATACCCCACGAATTCCCCGGGTACAACACACACACCA
TCTTTCCCCTCTCCCCGCGTCAGTCACCTTGCATCACACCGAGACATCCAGCTGATCCTC
CCGCGACACCATTCTCCCGCCCCTCTCTCTCCCCTCGATCGCCGGCCGGCCGAAACCATC
CATCCTTCCATGGGCTCCAAACCCGCGGACGCGGCAGGGAGCCGGCAGCAGCAGCAGGTG
AAGGAGGAGTCGTCCATGGCGGACGTGTACAACCACGAGCTGACGCCGCTGCAGAAGCAC
GCCGCCTTCTTCGACCGGAACAGGGACGGCGTCATCTACCCCTCCGAGACCTACCAAGGG
CTGCGCGCCATCGGCTGCGGCGTCGTCGTGTCCGCCGCCGGCGCCGTCTTCATCAACGGC
CTCCTCGCGCCCAAGACGGTACCGGCGAACGTGAACCCCCAAGCTTTCAAGTTCCCCAT
CTACGTAAAGACCATTCAGCAGGGCAAGCATGGGAGTGATACAGACGTGTACGACACCCA
GGGAAGGTTTGTTCCTGAAAAGTTTGAGGAGATATTCAAGAAGCATGCCCACACTAGGCC
TGATGCCCTAACGGACAAAGAGCTTGGGGAGATGCTTAAAGCAAACAGGGATCCTAAAGA
TTTCGCTGGACGGGTGGGCGCTTTCGTAGAGTGGAGACTTCTCTACGCGCTGTGCAAAGA
CAAGGAGGGATTTCTTCACAAGGAGACTGTCAAGGCGGTCTATGATGGCAGCGTGTTTGA
GAAGTTGGAGCGAGAAAAGAAGGAAGCTAAGGAATTTGCCAAGAAGAAATGATGAAATGT
CCTCCGATGCCCACTCATTTGTGGTTCTGTACAATTATCTGTAGATTGGGGTGTGCTTGT
GAGTTCCGTTCCCGAAATTATTGTTGTGGTCGTTTGGTTTCATTTGTTTCTGGTTTCATG
CCTCCTGGAATGAGGCTGGGAGCCTGATTGTAAATGTATAGAAACTGCTTGCAAATTGGG
AAAAAACATTCACATTTGGTTTC

>Clo8-B [organism=Triticum aestivum] EF-hand calcium binding protein, caleosin mRNA, full CDS

CTCCCGCTTTCCTCTCCCCGCGCCACCTTGGCACCATTACCGATATACCCAGCTGATCTT
CACCGCTTTCCTGCCCCTCGATCGCCGGCCGGCCGAAACCATCCATCCATCCATGGGCTC
CAAACCCGCGGACGCCGCAGGGAGCCAGCAGCAGCAGGGGGATGAGTCGTCCATGGCGGA
CTTGTACAGCAGCCACGAGCTGACGCCGCTGCAGAAGCACGCCGCCTTCTTCGACCGGAA
CAAGGACGGCATCATCTACCCCTCCGAGACCTACCAAGGGCTACGCGCCATCGGCTGCGG
CGTCGTGTTGTCCGCCGCCGGCACCGTCTTCATCAACGGCTTCCTCGCGCCCAAGACGGT
ACCGGCGAACGTGAAGCCTCCAGCTTTCAAGTTCCCCATCTACGTAAAGACCATTCAGC
AGGGCAAGCATGGGAGTGATACAGACGTGTACGACACCCAGGGAAGGTTTGTTCCTGAAA
AGTTTGAGGAGATATTCAAGAAGCATGCCCACACTAGGCCTGATGCCCTAACGGACAAAG
AGCTTGGGGAGATGCTTAAAGCAAACAGGGATCCTAAAGATTTCGCTGGACGGGTGGGCG
CTTTCGTAGAGTGGAGACTTCTCTACGCGCTGTGCAAAGACAAGGAGGGATTTCTTCACA
AGGAGACTGTCAAGGCGGTCTATGATGGCAGCGTGTTTGAGAAGTTGGAGCGAGAAAAGA
AGGAAGCTAAGGAATTTGCCAAGAAGAAATGATGAAATGTCCTCCGATGCCCACTCATTT
GTGGTTCTGTACAATTATCTGTAGATTGGGGTGTGCTTGTGAGTTCCGTTCCCGAAATTA
TTGTTGTGGTCGTTTGGTTTCATTTGTTTCTGGTTTCATGCCTCCTGGAATGAGGCTGGG
AGCCTGATTGTAAATGTATAGAAACTGCTTGCAAATTGGAAAAAAACATTCACATTTGGT
TTC

>Clo8-D [organism=Triticum aestivum] EF-hand calcium binding protein, caleosin mRNA, full CDS

CGCCTCTCCCCGCGTCAGTCACCTTGGCATCACATCACACCGAGACATCCAGCTGATCCT
CCCGCGACACCGTTCTCCCGCCCCCCTCTCTCCCTCTCCTCGATCGCCGGCCGGCCGAAA
CCATCCATCCACCCATGGGCTCCAAACCCGCGGACGCCGCAGAGAGCCAGCAGCAGCAGG
TGAAGGAGGAGCAGTCCTCCATGGCGGACGTGTACAACCACGAGCTGACGCCGCTGCAGA
AGCACGCCGCCTTCTTCGACCGGAACAGGGACGGCATCATCTACCCCTCCGAGACCTACC
AAGGGCTGCGTGCCATCGGCTGCGGCGTCGTCGTGTCCGCCGCCGGCGCCGTCCTCATCA
ACGGCCTCCTCGCGCCCAAGACCGTACCGGCGAACGTGAAACCTCCGGCTTTCAAGTTCC
CAATCTACGTGAAGACCATCCAGCAGGGCAAGCATGGGAGTGACACAGACGTGTACGATA
CCAACGGAAGGTTCGTTCCTGAAAAGTTTGAGGAGATATTCAAGAAGCATGCCCACACCA
GGCCTGATGCCCTAACGAGCAAAGAGCTGGGGGAGATGCTTAAAGCAAACAGGGACCCTA
AAGATTTCAAAGGACGGGTGGGCGCCTTCGGTGAGTGGAGACTTCTCTATGCACTGTGCA
AAGACAAGGAGGGGTTTCTTCACAAGGAGACTATGAAGGCGGTCTATGATGGCAGGGTGT
TTGAAAAGTTGGAGCGAGAAAAGAAGGAAGCTAAGGAATTTGCCAAGAAGAAATGATGAA
ATGTCCTCCGATACCCGCTTATTTGTGGTCCTGTGCAAAGTACTTGTAAATTGGGGTGTG
CTTGTGAGTTCGGTTCCTGAAATTAATGTTGTGATCGTTTGGTTTCATTTGTTATAGGTT
TCATGTTTCTTGGAATGAGAATGGGAGCCTGATGTAAAT

>Clo9-A [organism=Triticum aestivum] EF-hand calcium binding protein, caleosin mRNA, full CDS

AGATCCTGCAAGCTCGGCTCGTCGCGAGTAACCGCCGCCATGTCGTCGTCCGATCCGTCGCTGGCCACCGAGGCGCC
CCAGGCGGCCGTCACCAGCGAGCGGAGGCTCAACCCCGACCTCCAGGAGCAGCTCGCCAAGCCATATCTGGCCAGAG
CAATGTCGGCGGTTGACCCGAGCCACCCGGAGGGCAGCAAGGGTCGGGACAGCAAGGGCATGAGCGTGCTCCAGCAG
CACGCCGCCTTCTTCGACCGCAACGGCGACGGGGTCATCTACCCATGGGAGACCTTCCAAAGGCTCCGAGCAATCGG
GCTCGGGTCCCCTTCAGCCTTCGGAACATCCATACTCCTCCACCTCGTCCTCACTTATCCTACTCAACCGGGATGGA
TGCCTTCCCCTCTGCTGTCGATCCATATAAAGAACATCCACAGGGGCAAGCACGGGAGCGACTCTGAGACGTATGAC
ACAGAAGGGAGGTTTGAACCAGCGAAATTCGATGCTATATTCAGCAAGTTTGGCAAAACTCGGCCAAATGCTTTGTC

AGAAGATGAGATTAACGCCATGCTTAAACACAACCGCAATATGTATGATTTCCTGGGCTGGGCCGCAGCCAACCTCG
AATGGAAGCTGCTGCACAAAGTGGCAAAGGATAAAGAAGGCTTTTTGCAGCGAGAAATCGTGAGGGGCGCCTTCGAT
GGCAGCCTGTTCGAGCGCCTGCAGGAGAGCAAGAAATCTACCTGAATGTGGCAGTGAGCCGCGTGGAGTCGTCTATA
AAACATCTGCACTGGAAATTTGGAAAGCCTCCCTGCATTGCAGTTTGAATTGTGTACGTGAAATAAGAAGTGTTTGG
CCTGTCTACGGCGGCCTAAACATCTTCATTTGTATTGTCATTTTGAACTGGAATTTGTACTGTTATTGGTAGCAGAC
GGAAGGTGTACAGTGGGACTTTGTAATGTGCACTCTTGAATCATCATTACCTTGCATAATATAGGCGTGT

>Clo9-B [organism=Triticum aestivum] EF-hand calcium binding protein, caleosin mRNA, full CDS
GGTTGGATTCCCTCTCTCTCTCTCTCAGCAGCACTGCTCTGAAGCTCTCTTCTTCTGGGC
CGGGGTTCACTCACGGACTCACAGTAACCCACAGTTCACAGATTCATTCGCTTGTTCGAT
CTGCAGATCCTGCAAGCTCGGCTCGTCGCGAGTAACCGCCATGTCGTCGTCGTCGTCCGA
TCCGTCGCTGGCGACCGAGGCGCCCCAGGCGGCCGTCACCAGCGAGCGCAAGCTCAACCG
CGACCTGCAGGAGCAGCTCGCCAAGCCATATCTGGCCAGAGCAATGGCGGCGGTTGACCC
GAGCCACCCGGAGGGCAGCAAGGGGAGGGACACCAAGGGCATGAGCGTGCTCCAGCAGCA
CGCCGCCTTCTTCGACCGCAACGGCGACGGGGTCATCTACCCATGGGAGACCTTCCAGAG
CCTGCGAGCAATCGGGCTTGGGTCGCCTTCAGCCTTCGGAACATCCATACTCCTCCACCT
CGTCCTCACTTATCCTACTCAACCGGGATGGATGCCTTCCCCTCTGCTGTCGATCCATAT
AAAGAACATCCACAGGGGCAAGCACGGGAGCGATTCTGAGACGTATGACACAGAAGGGAG
GTTTGAACCAGCGAAATTCGATGCTATATTCAGCAAGTTTGGCAAAACTCGGCCAAATGC
TTTGTCAGAAGATGAGATTAACGCCATGCTTAAATACAACCGCAATATGTATGATTTCCT
GGGCTGGGCCGCGGCCAACCTCGAATGGAAGTTGCTGCACAAGGTGGCAAAGGATAACGA
AGGCTTTTTGCAGCGAGAAATCGTGCGGGGCGCCTTCGATGGCAGCCTGTTCGAGCGTCT
GCAGGAAAGCAAGAAATCTACCTGAATGTGGCAGTGAGCCTCGTCTCTAAAATATCTGCA
CTTGAAATTTGAAAAGCCTCCCTGCATTGTAGTTTGAGTTGTGTACGTCAAATAAGAAGT
GTTTGGCCCGTCTGCGGCGGCCTAAACATTTTCATTTGCATTGTTATTTTGAACTGGAAT
TTGTATTTTTATTGGTAGCGCACAGAAGGTGTACAGTGGAACTTGTAATGTGCACTCGTG
AATCATCATTACCTCGCACTATTATAGGCGTGGTTTCTTTCTTTTTACC

>Clo9-D [organism=Triticum aestivum] EF-hand calcium binding protein, caleosin mRNA, full CDS
AGATCCTGCGAGCTCGGCTCGTCGCGAGTAACCGCCGCCATGTCGTCGTCCGATCCGTCG
CTGGCGACCGAGGCGCCCCAGGCGGCCGTCACCAGCGAGCGCAAGCTCAGCCCCGACCTG
CAGGAGCAGCTCGCCAAGCCATATCTGGCCAGAGCAATGGCGGCGGTTGACCCGAGCCAC
CCGGAGGGCAGCAAGGGTCGGGACACCAAGGGGATGAGCGTGCTCCAGCAGCACGCCGCC
TTCTTCGACCGCAACGGCGACGGGGTCATCTACCCATGGGAGACCTTCCAAAGCCTCCGA
GCAATCGGGCTTGGGTCGCCTTCGGCCTTCGGAACATCCATACTCCTCCACCTCGTCCTC
ACTTATCCTACTCAACCGGGATGGATGCCTTCCCCTCTGCTCTCGATCCATATAAAGAAC
ATCCACAGGGGCAAGCACGGGAGCGACTCTGAGACGTATGACACTGAAGGGAGGTTTGAA
CCAGCGAAATTCGATGCTATATTCAGCAAGTTTGGCAAAACTCGGCCGAATGCTTTGTCA
GAAGATGAGATCAACGCCATGCTTAAACACAACCGCAATATGTATGATTTCCTGGGCTGG
GCCGCAGCCAACCTCGAATGGAAGCTGCTGCACAAAGTGGCAAAGGATAAAGAAGGCTTT
TTACAGCGAGAAATCGTGAGGGGCGCCTTCGATGGCAGCCTGTTCGAGCGCCTGCAGGAG
AGCAAGAAATCTACCTGAATGCGGCAGTGAGCCGTGCGCAGTCCAGACCGAGCGTCGTCA
ATGAAACATCTGCACTTGAAATTTGAAAAGCCTCCCTGCATTGTAGTTTGAGTTCTGCAC
GTCAAATAAGAAGTGTTTGGCCCGTCTACGGCGGCCTAAACATTTTCATTTGTATTGTTA
TTTTGAACTGGAATTTGTACTGTTATTGGTAGCGCACACAAGGTGTACAGTGGAACTTTG
TAATGTGCACTCGTGAACCATCAGTACCTCGCGTAATATAGGCGTGTCCGAAAATCAAAA
ACCTGCCCGGGCGGCCGCTCGAA

>Clo10-A [organism=Triticum aestivum] EF-hand calcium binding protein, caleosin mRNA, full CDS; Derived from WSS

GCACAGAGGGTTTCATGGCGGAGGAAGCTAAGGCGCCCGCCGACGCAACGTCGACGGTGG
CGGAGGCGGCGCCGGTGACGGCCGAGCGGCCGGTCCGGGCGGACCTGGAGACACACCTCC
CAAAGCCCTACTTAGCTCGAGCTCTGGTCGTGCCGGATGTGTACCATCCTGAAGGAGAGA
GCGGGGAGGGGCACGAGCACGGGCAGAAGAGCGTGCTTCAGCAGCATGTCGCCTTCTTCG
ACCTGGACGGCGACGGCATCGTCTATCCCTGGGAAACTTACGGAGGAATGAGGGCATTGG
GCTTCAACGTGATCGTGTCGTTCGTATTCGCAATTGTCTTTAATATTGGTTTCAGCTTCC
CAACTCTACCTAGCTGGATACCATCTCCCTTGTTGCCCGTATACATCAAGAACATCCACA
GGGGTAAGCATGGTAGCGATTCCTCAACTTATGACACAGAGGGAAGGTTCATGCCGGTAA
ATTTCGAGAGCATCTTCAGCAAGTATGCCCGCACGGCGCCGGACAAGCTTAGCTTCGGCG
ACATCTGTAGGATGACCGAAGGCAACCGGCTGAACTTCGACTTCATTGGATGGATTGTGA
GCAAGGGGGAGTGGATACTGCTGTACGTGCTTGCAAAGGACGAGGAGGGCTTCCTCTCCA
GAGAAGCTGTTCGCCGCTGCTTTGATGGTAGCCTGTTCGAGTTCATCGCCCAGCAAAGGA
GGGAAGCCCACGACAAGCAGCACTAGAGTTGCAACGCTGTCATCTTCGTGAGTAGGACTG
TAGACCAGATGGATTACCTCATGTATGCCTAATATATGAAATAAGAGAGGAACGAGTATG
AAGTTCAGAGAATAAAGTTGTACACTTCTCAACTAAACTTGAGAACATAAGTACTCTGCA
CTGTGGT

>Clo10-B [organism=Triticum aestivum] EF-hand calcium binding protein, caleosin mRNA, full CDS

CGCGAGATCAAGCCGAAAAGTGTGGTGCACGCGCACAGAGGGTTATGGCGGAGGAAGCTA
AGGCGCCCGCCGACGCAATGTCGACGGTGGCAGAGGAGGCGCCGGTGACGGCCGAGCGGC
CGGTCCGGGCAGACCTGGAGACACACCTCCCAAAGCCCTACTTGGCTCGAGCTCTGGTCG
TGCCGGATGTGTACCATCCTGAAGGAGAGAGCGGGGAGGGGCACGAGCACGGACAGAAGA
GCGTGCTGCAGCAGCATGTGGCCTTCTTCGATCTGGATGGCGACGGCATCGTCTATCCCT
GGGAAACTTACCGAGGAGTGCGGGCATTGGGCTTCAACGTGATCGTGTCGTTCGTGTTGG
CAATTGTCTTTAATATTGGTTTCAGCTTCCCAACTCTACCTAGCTGGATACCATCTCCCT
TGTTGCCCGTATACATCAAGAACATCCACAGGGGTAAGCATGGTAGCGACTCCTCAACCT
ATGACACAGAGGGAAGGTTCATGCCGGTAAATTTCGAGAGCATCTTCAGCAAGTATGCCC
GCACGGCGCCGGACAAGCTTAGCTTCGGCGACATCTGGAGGATGACCGAAGGCAACCGGC
TCAACTTCGACTTCATTGGATGGATTGTGAGCAAGGGGGAGTGGATACTGTTGTACGTGC
TTGCCAAGGACGAGGAAGGCTTCCTCTCCAGAGAAGCTGTTCGCCGCTGCTTCGATGGTA
GCCTGTTCGAGTTCATCGCCCAGCAAAGGAGGGACGCCCACGACAAGCAGCACTAGAGCG
CAACGCTGTCATCTTCATGAATAAGAGAGGAATGAGTACGAAGTTCGCAGAATAAAGTTG
TACACTTCTCAACTAACTCGAGAACA

>Clo10-1-D [organism=Triticum aestivum] EF-hand calcium binding protein, caleosin mRNA, full CDS

AGGCACAGAGGGTTTTATGGCGGAGGAAGCTAAGGCGCCCGCCGACGCAACGTCGACGGT
GGCGGAGGAGGCGCCGGTGACGGCCGAGCGGCCGGTACGGCCAGACCTGGAGACACACCT
CCCAAAGCCCTACTTGGCTCGAGCTCTGGTCGTGCCCGATGTGTACCATCCTGAAGGGGC
CGTGGAGGGGCACGAGCACGGGCAGAAGAGCGTGCTGCAGCAGCATGTGGCCTTCTTCGA
CCTGGATGGCGACGGCATCGTCTATCCCTGGGAAACTTATGGAGGAATGAGGGCATTGGG
CTTCAACGTGATCGTGTCGTTCGTATTCGCAATTGTCTTTAATATTGGTTTCAGCTTCCC
AACTCTACCTAGCTGGATACCGTCTCTCTTGTTGCCCGTATACATCAAGAACATCCATAG

GGATAAGCATGGTAGCGATTCCTCAACTTATGACACAGAGGGAAGGTTCATGCCGGTAAA
TTTCGAGAGCATCTTCAGCAAGTATGCCCGCACGGCGCCGGACAAGCTTAGCTTCGGCGA
CATCTGGAGGATGACCGAAGGCAACCGGCTTAACTTCGACTTCATTGGATGGATTGTGAG
CAAGGGAGAGTGGATACTGTTGTACGTGCTTGCCAAGGACGAGGAAGGGTTCCTCTCCAG
GGAAGCTGTTCGCCGCTGCTTCGATGGTAGCCTGTTCGAGTTCATCGCCCAGCAACGGAG
GGAAGCCCACGAGAAGCTGCACTAGAGTGCAATTCTGTCATCTTCATGAGTACGACCAGA
TCGATTGCCTCATGTATGCCTATGAAATAAGAGAGGAACGAGTACAAAATTCAGAGAATA
AAGTTGTACACTTCTCAACTAAACTTGAGAACATAAGTACTCTGTACTGTGGTGTG
A

>Clo10-2-D [organism=Triticum aestivum] EF-hand calcium binding protein, caleosin mRNA, full CDS
GGGTTATGGCGGAGGAAGATAAGGCGCCCAGCGACGCAATGTCGACGGTGGCGGAGGAGG
CGCCGGTGACGGCCGAGCGGCCGGTCCGGGCAGACCTGGAGCCGCGCCTTCCCAAGCCAT
CAGACTTGGCTCGAGCTCTGGTCGTGCCGGATGTGTACCATCCTGAAGGAGAGAGCGGGG
AGGGGCACGAGCACGGCCAGAAGAGCGTCCTGCAGCAGCATGTGGCCTTCTTCGACCTGG
ACGGCGACGGCATCGTCTATCCCTGGGAAACTTACCGAGGAATGAGGGCATTGGGCTTCA
ACGTGATCGTGTCGTTCGTGTTGGCAATTGTCTTTAATATTGGTTTCAGCTTCCCAACTC
GACCTAGCTGGATACCGTCTCCCCTGTTGCCCGTACACATCAAGAACATCCACAGGGGCA
AGCATGGTAGCGATTCCTCAACTTATGACACAGAGGGAAGGTTCATGCCGGTAAATTTCG
AGAGCATCTTCAGCAAGTATGCCCGCACGGCGCCGGACAAGCTTAGCTTCGGCGACATCT
GGAGGATGACCGAAGGCAACCGGCTGAACTTCGACTTCATTGGATGGTNNGTGAGCAAGG
GGAAGTGGATACTGCTTTACGTGCTTGCCAAGGATGAGGAAGGCTTCCTCTCCAGAGAAG
CTGTTCGCCGCTGCTTTGATGGTAGCCTGTTCGAGTTCATCGCCCAGGAAAGGAGGGAAG
CCCACGAGAAGCTGCACTAGAGTGCAACGCTGTCATCTTCGTGAGTAAAACTGTAGACAG
ATGTATTACCTCATGTATGCCTATGAAATAAGAGAGGAACGAGTACGAAGTCCAGA

>Clo11-A [organism=Triticum aestivum] EF-hand calcium binding protein, caleosin mRNA, full CDS; Derived from WSS
GCGGGTTATGGCGGAGGAAGATAAGGCGCCCAGCGACGCAACGTCGACGGTGGCGGAGGA
GGCGCCGGTGACGGCCGAGCGGCTGGTCCGGGCAGACCTGGAGCTGCGCCTTCCCAAGCC
ATACTTGGCTCGAGCTCTGGCCGTGCCCGATGTGTACCATCCTGAAGGAGAGAGCGGGGA
GGGGCACGAGCACGGCCAGAAGAGCGTGCTGCAGCAGCATGTGTCCTTCGACCTGGACGG
CGACGGCATCGTCTATCCCTGGGAGACTTACCGAGGAATGCGGTCGCTGGGCTTCAACGT
GATCTTGTGTTTTATAGCGGCAATTGCCATAAATATTGGTCTAAGCTTCCCAACTCTACC
TAGCTGGATACCATCTCCCTTGTTCCCCATACACATCAAGAACATCCACAAGGATAAGCA
TGGCAGCGACTCCTCGACGTACGACACGGAGGGAAGGTTCATGCCGGTAAATTTCGAGAG
CATCTTCAGTAAGTACGCCCGCACGGCGCCGGACAAGCTCAGCTTTGGCGACATCTGGAG
GATGACCGAAGGCAACAGGCTGCAGTATGACTTCTTTGGGTGGTTGGTGAGCAAGGGAGA
GTGGATACTGCTGTACGTGCTTGCCAAGGACGAGGAGGGCTTCGTCTCCAGAGAAGCTGT
TCGCCGCTGCTTCGATGGTAGCTTGTTCGAGTTCATCGCCCAGCAAAGGAGGGAAGCCCA
CGAGAAGCAGCACTAGAGTCACAGGACTGAGTAGAGTGCAACGTTGTCATCTCGGAGTGT
CTTCGATCGTGAGTTTTCAAATGTGGACCAGATTGATTACCTCATCATGTATGCCTAGGA
AATAACGACGAACGAATACCAAGTTTAGAGGGCTGAAAAGTTGTACACTTTTATATAATA
ATAATAAGTTGTAAACATATCAATTATAAACTCGAGGACACAAGTATTTTGGTGGGCCCT
TTGCTCCTTCAGTTTCTTCCAACGGCAA

>Clo11-B [organism=Triticum aestivum] EF-hand calcium binding protein, caleosin mRNA, full CDS
TTGCTTTCGATTCCGGCGCGCGCGGGTTATGGTGGAGGAAGATAAGGCGCCCAGCGACGC
AATGTCGACGGTGGCGGAGGAGGCGCCGGTGACGGCCGAGCGGCCGATCCGGGCAGGCCT
GGAGCCGCGCCTTCCCAAGCCATACTTGGCTCGAGCTCTGGTCGTGCCTGATGTGTACCA
TCCTGAAGGAGAGAGCGGGGAGGGGCACGAGCACGGGCAGAAGAGCGTGCTTCAGCAGCA

TGTGTCCTTCTTCGACCTGGACGGCGACGGCATCGTCTATCCATGGGAGACTTACGGAGG
AATGCGGTCGCTGGGCTTCAACGTGATCTTGTGTTTTATAGCGGCAATTGCCATAAATAT
TGGCCTAAGCTTCCCAACCCTACCTAGCTGGATACCATCTCCCTTGTTCCCCATACACAT
CAAGAACATCCACAAGGATAAGCATGGCAGCGACTCCTCGACCTACGACACGGAGGGAAG
GTTTATGCCGGTAAATTTCGAGAGCATCTTCAGCAAGTACGCCCGCACGGCGCCGGACAA
GCTCAGCTTCGGCGACATCTGGAGGATGACCGAAGGCAACCGGCTGCAGTATGACTTCTT
TGGATGGTTGGTGAGCAAGGGAGAGTGGATACTGCTGTACGTGCTGGCCAAGGACGAGGA
GGGCTTCGTCTCCCGAGAAGCTGTTCGCCGTTGCTTTGATGGCAGCTTGTTCGAGTTCAT
CGCCCAGCAAAGGAGGGAAGCCCACGAGAAGCAGCACTAGAGTCACAGGACTGAGTAGGG
TGCAACGCTGTCATCTCGGAGCGTCTTCGATCGTGAGTATTCAAATGTGGACCAGATTGA
TTACCACATCATGTATGCCTAGGAAATAGAGACGAACGGATACCAAGTTCAGAGGGCTGA
AAAGTTATACACTTTTATATAAAAAAAATAAGATGTATACATAAACTCGTGGACACAAGT
ATTTTGGTGTCCTTTGCTCCTTTACTTCGAACGGCAATGAGGGCATGTACTATGGTGGCA
TCACCAAGGTACCGAGCGATGTATTCCACATGGACTGTTTTC

>Clo11-D [organism=Triticum aestivum] EF-hand calcium binding protein, caleosin mRNA, full CDS
ACAGAGAGAAGCGCGGGTTATGGCGGAGGAAGATAAGGCGCCCAGCGACGCAATGTCGAC
GGTGGCGGAGGAGGCGCCGGTGACGGCCGAGCGGCCGGTCCGGGCAGACCTGGAGCCGCG
CCTTCCCAAGCCATACTTGGCTCGAGCTCTGGCCGTGCCCGATGTGTACAATCCTGAAGG
GGCCGTGGAGGGGCACGAGCACGGGCAGAAGAGCGTGCTGCAGCAGCATGTGGCCTTCTT
CGACCTGGACGGCGACGGCATCGTCTATCCCTGGGAGACTTACGGAGGAATGCGGTCACT
GGGCTTCAACGTGATCTTGTGTTTTATAGCGGCAATTGCCATAAATATTGGTCTAAGCTT
CCCAACTCTACCTAGCTGGATACCATCTCCCTTGTTCCCCATACACATCAAGAACATCCA
CAAGGATAAGCATGGCAGCGACTCCTCGACCTACGACACGGAGGGAAGGTTTATGCCGGT
AAATTTCGGGAGCATCTTCAGCAAGTACGCCCGCACGGCGCCGGACAAGCTCAGCTTCGG
CGACATCTGGAGGATGACCGAAGGCAACCGGCTGCAGTATGACTTCTTTGGATGGTTGGT
GAGCAAGGGAGAGTGGATACTGCTGTACGTGCTGGCCAAGGACGAGGAGGGCTTCGTCTC
CAGAGAAGCTGTTCGCCGCTGCTTCGATGGTAGCTTGTTCGAGTTCATCGCCCAGCAAAG
GAGGGAAGCCCACGAGAAGCAGCACTAGAGTCACAGGACCGAGTAGAGTGCAACGCTGTC
GTCTCGGAGCGTCCTCGATCGTGAGTGGACCAGATTGATTACCTCATCATGTATTCCTAG
GAAATAAGAGACGAACGAATACCAAGTTCAGAGGGCTGAGAAGTTATACACTTTTATATA
AAAATAAGTTGTACACGTATCAATTATAAACT

2) Rye (*S. cereale*)

>Clo1 [organism=Secale cereal] EF-hand calcium binding protein, caleosin mRNA, full CDS
CACGGCTTGCCCGCAAGTTATCTCCTCAACGATTCCGCTCACGGTTCATCGGCTGCCACC
CAGTTCTGGTGCGCCGGCGAAGAGCGGCGAAGATGGCCACCAAGGGGCGCAAGGTCGAGG
TCCGTGACGCGAGCAGGACGGACGGGAAGGGCGACGCCGCGGACGTGCACGTCGTCCGCG
AGTCGGCCAGGACCGAGGGGAAGAGCGACCACGACACCGCCGGCGGCGCCGGGCTCCATG
GCGCGAGCATGACGGATGGGCACGGCGACGCGGGCGGCATGGTCGGGGACCTCCGGGGCA
TCGGGGGGAATGACTCCCTGAAGATCGTCGCGATGCAGGCGCCGGTCACCGTGGAGCGCC
CCGTCCGCGGCGACCTCGAGGAGCACCTCCCCAAGCCCTATTTGGCGAGGGCGCTGGCGG
CTCCGGACATGTACCACCCGGAGGGGACGACGGACGATCACCACCACCACAACATGAGCG
TGCTGCAGCAGCACGTCGCCTTCTTCGACCGCGACAACAACGGCATCATCTATCCCTGGG
AGACATACGATGGCTGCCGTGCGGTTGGGTTCAACGTGTTCATGTCCGCCTTCATCGCGT

TTCTGGTGAACCTGGTCATGAGCTATCCCACCCTGCCCGGCTGGCTGCCGAACCCCCTCT
TCCCGATTTACGTCCACAACATCCACAAGAGCAAGCACGGGAGTGACTCGGGGACCTATG
ACAAAGAGGGGCGGTTCATGCCGGTGAACTTCGAGAACATCTTCAGCAAGTACGCCCGCA
CGTACCCGGACAGGCTCAGCTACAGGGAGTTGTGGCGGATGACCGAGGGGTCGCGGGAGG
TGTTCGATTTCTTCGGCTGGGTGGCGATGAAGCTGGAGTGGTCGTTCCTGTACGTGCTGG
CTCGGGACGACGAGGGGTACCTGTCGAGGGAGGCCATCCGGCGCATGTACGACGGCAGCC
TCTTTGAGTACATGGAGAGGCAGCGCATGGAGCACGTCAAGATGTCCTAAGATCACCGGC
TCGTCGTCGATGGTGGATCTCGCAGGGTCGGAGCTGCACCCACCTGTGATGCTTTCCGTG
CCCGTATGCGTATGTACAGCAGGCCACAGATCCATGTGGTGTGCCGCTGGTGTGGTGAAA
CATAAATAACGTGATATGCCATGCTTGTATTGTACAGTATATATGTTTAGCCCCGTCCGG
TCTCCCTCTCTTCCTCCGGCCGGACCGTCGACTGCAGA

>Clo3 [organism=Secale cereal] EF-hand calcium binding protein, caleosin mRNA, full CDS
GGGTTACCGGCGGCAGCGGCGGCAGCAGCGATGGCGATCCGGCGACAATCATCAGCAGCAGCTTCTCTACTCCTGCC
CCCGGTGGCTGCTCTTCTGTTTCTATGGGTGTTTGGCGGGGGGCATGTGATGGCACATAATGAGATTCGCAACATGA
CGGCACTCCAGAAACATGTCTCCTTTTTCGACCGTAACAAGGATGGCATCATTACTCCTTCGGAAACATTTGAAGGG
TCTGTCGCAATTGGTTTTAATGTTACATATGCCAGAGAATTTGCCACCTTGGTGCATACTGCTAATGGTCCTATAAC
AAGCCCCGCTGATGCACCATTGCCTCACTTATCAATATACATAGAGAATATGCAGAGAGGAATGCATGGGAGTGATA
CCGGTGCATTTGATGTTAAAGGAAGGTTTGTTCCACAAAAGTTTGAGGAAATATTCATAAAGCATGCAAAAACTAGA
CCAGATGGTTTGACATATTTGGAGGTGGAGGATATGATCCTAGCAAATCGAGATCCACTGGACCCTGCATCATGGGA
GGGACCTCAAATAGAATGGGGCGGAATATACAACGTCGCGAGTGACAATGATGGATTTCTTCACAAGGACGATGCGA
GAGGTATATATGACGGAAGTGTGTTTGTAAAGCTGGAGGAAAAGAGGGCCTTTTCTCATCATAGTGCAATGTAATAG
AGTGCGCAACATGTTGTACGCTGAAATAATTAGGGGAACACATAGTGTGTGTACTAAGACTGGTATATATTTGTTCA
AGTGTTTGTTTGCACATAAGTAAAAT

>Clo4 [organism=Secale cereal] EF-hand calcium binding protein, caleosin mRNA, full CDS
ATCGTTGCAGGCGACGATGGCCGGTCATCGACAATCGCTGGCAGTGGCTTCTCTGAAGCT
CCCAGTTCTTCTGCTTCTGTGGATCTTTAGCTTGAACTGGGGGCATGCCGTGGCGCACTT
TGATCCTGCAAACATGACGGAGCTCCAGAAACATGTCTCCTTTTTCGACCGCAACAAGGA
TGGCTTCATCACTCCTGCGGAAACCATCCAAGGGTTTGTTGCAATCGGTTGCGAGTATGC
ATTTGCTACTGCTGCCTCAGCCTCCATTCACGGTGCCCTTGCTCCTCAAACAACCCCGGC
TGGTACACCACTGCCTCACTTGACAATATACGTGGAGAATATCCACAAAGCTATGCATGGAAGTGATTCGGGTGTAT
ATGATGCTAAAGGAAGGTTTCTTCCCCAAAAGTTTGAGGAATTATTCAAGACATATGCAATACTTCGTCCAGATGCA
TTGACACTTACAGAGATGCATGCGATGCTCTTTGCTAAACGAGATCTAGACCCGATTTCATGGGCGCCACCCGAGAT
AGAGTGGGGGCTATTATTCACGCTTGCAAGCGATTGGCTTGGATTTCTTCACAAGGACAGTG
TTAGAGGTGTATACGATGGAAGTGTGTTTACCAAGTTGGAGAAGAAATGGCACCCTTCTC
AAAGTGATATATGATGAATTTGATGTAATGTGGGACCCACATACAAGCGACGGAGAAGGC
CCAATTCATCCACACCTGGAGTATTAGTTTAGAGTAAGAGTTTGGATATGGAAAGGTTCG
TCCCGAAGAAGGTTTTCCTGCTATCTCCAAATTCAACTAGAGTTTTCCCCCTCCAAGTTG
TAACTTGCTTTATAAGCCATGAAGAGTCGATAATATGATCGCCAAGTTGAATACATTTCC
CTACTGTGTCTTCTCTCTCATGCGCCCTTTGTTATTAGTACGCCCTAGGTGCCTATCTAG
CCTCGTCCACCAGTTGACCACGGCTACTGTTGTAAATATTCCTTACACGCGATATTGTGA
TAATAATATTTCTGTATGCATTTACGAGTTAAAAAAAAA

>Clo5 [organism=Secale cereal] EF-hand calcium binding protein, caleosin mRNA, full CDS

GGTATCAACGCAGAGTACGATGGGGACTCCACGCGAGCCGCGGCAAAGACACGGAGAAGAGAGGGACAACAGTCAAA
AGCGAGCGAGGCGGAAGAAATCCCTTTGAGATGGACGCGCGACCGCGACGGGCATCGTCCTCGCCGCCGCCAGCGGC
GGCGGCGGCCTTGTCCCTGCTGCTCCTGTTCCCCATGTTCTTAGGGAGCCAGGCGTCGGGTTACGGCGACGATTCGG
GCGCCGGCGGCATGACGGCGCTGCAGAAGCACGCGGCGTTCTTCGACGCCGACAAGGACGGCGTCGTCACCTTCTCC
GAGACATACGCAGCGTTTCGGGCCCTCGGATTCGGATATGGCGCCTCCACCTTGAGTGCTACCTTCATCAATGGCGT
CCTTGGCCCCCAGACCAGACCGGAAAACGATACGGCGCGCATGTCCATCTACATCGAGAACATCCACAAAGGCATCC
ATGGAAGCGATTCAGGCGCGTATGACTCCCAAGGAAGGTTCGTTCCCGAGAAGTTCGAGGCGGTGTTCGCCAGGCAC
GCCAAGACGGTGCCGGATGCCCTGACGTCCGGTGAGGTGGACGAGCTGATCACCGCGAACCGGCAGCCCAGCGACTA
CGCAGGATGGGCGGGCGCGTCGGCGGAGTGGAAGTTGCTGTACAGCATCGGCAAGGACAAGGACGGGCTCCTCCGCA
AGGACGCCGCCAGGGGCGTCTACGACGGCAGCCTCTTCGCCAGGGTGGTGCACGAGCGGAGGGCGGCGCGGGAAGAA
ACCCAGGCATGATCGATGGATTTATTATCGTGTCACGCCGTCCGGACTGATGCGTCGCTGCCTTAGCTACAGACAGT
AGTACGTCGTGTACTTACTAATTAATTGGCCTGTGTGAACGGGTGTTACTAATTAATTGGCCAGTGTGAACGGGTGT
G

>Clo6 [organism=Secale cereal] EF-hand calcium binding protein, caleosin mRNA, full CDS
AAGCAGTGGTATCAACGCAGAGTACATGGGGACGCAGACGCAGCCTCGATCCGGGAAAAGAAGAGGATAGATCAGGA
GTCAGCTCGCCAGAGAGAAAGGGAGGGTCGAGAGAGGAGGGATCGCCTGACACGATGGGCGCCACCGGACAACGCCG
GCTGTCGTCTCTGCCCGCCGCGGTGGCCGTGTCTCTCCTGCTTCTGCTCGCCGTGTCCTTCGGGAGCCAGGCGGCGT
CGGCGGCGGCTCCGGCCTGGACGACGGACCTGGAGAAGCACGTGGCGTTCTTCGACACCAACAACGACGGCATCGTC
TCCTTCTCCGAGACGGAGCATGGGCTCCGTGCCATCGGTCTCGGAGCTCTCGAGGCCGCCGCCAGCGCCACCCTGAT
CAACGGAGTCATCGGGCCCAAGACCAGACCTGAAAATGCCACGACTTCGAGGTTTGACATCTACATAGCCAACATCC
ACAAAGGGATCCACGGGAGCGACAGTGGCTCGTACGATGCCCAAGGAAGGTTTGTCCCCGCCAAGTTCAACGAGATA
TTCACCAAGTACGCCAAGGCCAAGCCGAACGCACTCAACGAGGCCGAGCTGGAGGCGATGCGCACTGCCAACAGGAA
GGAGGGTGACTTCAAAGGATGGGCGGCGTCGAAGGCGGAGTGGGGCATGCTGTACAACCTCGCCAAGGACAAGGACG
GCTTCCTTCAGAAGGACACCGCGCGCGCCGTCTACGACGGCAGCCTCTTCGTTAAGCTAGCGAAGAAGAACGGTGCT
TCATCTGGAAATTAACCGGGCGTGATTTGTACCCCGTATTTTTCGAGATGAAATATGTACTCCGCGTTAATTATTCG
TGTCTTGGTCGTTGAGTATGTACTGTCACAGTGTCATTGTACAATAAAAATGGAATCTGTTTTTTT

>Clo7 [organism=Secale cereal] EF-hand calcium binding protein, caleosin mRNA, full CDS
CACCCCCGGCCTCACCACCTTGGCATCACTACTTCTCCCTCCC
CCATCTCCCCGCAACCCTCCCCCCCGTCTCGTTGGAATCCATGGCGTCCAAGTCCTCCGT
CACCGCAGGCGGGCAGCCCAAGGGCAAGGAGGAGGCGTCCATGGCGGACGTGTACAACCA
CGAGCTGACGCCGCTGCAGCGGCACGTCGCCTTCTTCGACCGGAACAAGGACGGCGTCAT
CCAACCCTCCGAGACCTACCAAGGGTTCCGCGCGATCGGGTGCGGCGTCGCGCTGTCCGC
CTTCAGCGCCGTCTTCATCAACGGCTTGCTCGGTCCCAAGACGGTCCCGGAGAACATGAA
GGTTGGAGCTTTCAAGTTTCCGATCTACGTAAAGAACATCCACAAGGGCAAGCATGGGAG
CGATTCGGGCGTGTACGATGCCAATGGAAGGTTTGTTCCTGAAAAGTTTGAAGAGATATT
CAAAAAGCATGCTCACACCAGGCCTGATGCCCTGACAGGCAAAGAACTGAATGAGTTGCT
TCAAGCAAACAGGGAGCCTAACGATTTGAAAGGACGAGTGGGTGGCTTCACGGAGTGGAA
AGTTCTCTACTCGCTGTGCAAAGATAAGGAGGGATTTCTTCACAAGGAGACCGTCAGGGC
AGTCTACGATGGCAGCCTGTTTGTGAAGTTGGAGCAAGAGAGGAAGCAAGCTAAGGAATC
TGCCAAGAAGAAATGATGAAAACTATCCCAATACCCTCTTATTTGTGATTGTGCGCAAGT
ATGCGTAAATTATGGTGTGCTTGCGAGTATGGTTCTGTAAATTAATAATGTCGTGATCTG
ATTTCGTGTTGTTTGTTTGTTCTTTTATGGAATGTGAATTCAAAGCCTATTTCTCTAGAT
ATATATCG

>Clo8 [organism=Secale cereal] EF-hand calcium binding protein, caleosin mRNA, full CDS

TCCATCCATCCATGGACACGGCAGGGAGCCACCAGCAGCAGCAGCAGCAGACGGAGGAGTCGTCCATGGCGGACGTG
TACGGCGGCCACGAGCTGACGCCGCTGAAGAAGCACGCCGCCTTCTTCGACCGGAACAGGGACGGCATCATCTACCC
CTCCGAGACCTACCAAGGGCTACGCGCCATCGGCTGCGGTGTCGTCCTGTCTGCCGCCGGCACCGTCTTCATCAACG
GCTTCCTCGCGCCCAAGACGGTACCGGCGAACGTGAAGCCTCCAGCTTTCAAGTTCCCCATCTACGTGAAAACCATT
CAGCAGGGCAAGCATGGGAGTGATACAGACGTGTACGACACCCAGGGAAGGTTTGTTCCTGAAAAGTTTGAGGAGAT
ATTCAAGAAGCATGCCCACACTAGGCCTGATGCCCTAACGGACAAAGAGCTGGGGGAGATGCTTAAAGCAAACAGGG
ATCCTAAAGATTTCGCTGGACGGGTGGGCGCTTTCGTAGAGTGGAGACTTCTCTACTCGTTGTGCAAAGACAAGGAG
GGATTTCTTCACAAGGAGACTGTCAAGGCGGTCTATGATGGCAGCGTCTTCGAGAAGTTGGAGCGAGAAAAGAAGGA
GGCCAAGGGATTTGCCAAGAAGAAATGATGAAATGTCCTCCGATGCCCACTTATTTGTGGTTCTCTGCAAGTATTAG
TAGATTGTGGTGTGCTTGTGAGTTCGGTTCCCGAAATTGTTGTTGTAATCGTTTGGATTCATGCTCATGGAATGAGA
ACGGGG

>Clo9 [organism=Secale cereal] EF-hand calcium binding protein, caleosin mRNA, full CDS

TTCCCTCTCAGCAGCATTGCTCCGAGTCTCTCTTCTCTTGGGTTCACTCACGGACTCACAGTAACCGACACTCCAGA
ACTCCAGATTCATTTGCTTTGTTCGATCTGCAGATCCTGCGAGCTCGGCTCGTCGCGAGTAACCGCCGCCATGTCGT
CGTCGTCGTCGTCCGACCCGTCGCTGGCGACCGAGGCGCCCCGGGCGGCCGTCACCAGCGAGCGGAAGCTCAACCCC
GACCTGCAGGAGCAGCTCGCCAAGCCATATCTGGCCAGAGCAATGGCGGCGGTTGACCCGAGCCACCCGGAGGGCAG
CAAGGGCAGGGACACCAAGGGCATGAGCGTGCTCCAGCAGCACGCCGCCTTCTTCGACCGCAACGGCGACGGGGTCA
TCCACCCATGGGAGACCTTCCAAAGCCTCCGAGCAATCGGGCTTGGGTCTCCTTCAGCCTTCGGAACATCCATACTC
CTGCACCTCGTCCTCACTTATCCTACTCAACCGGGATGGATGCCTTCCCCTCTGCTATCGATCCATATAAAGAACAT
CCACAGGGGCAAGCACGGGAGCGATTCTGAGACGTACGACACTGAAGGGAGGTTTGAGCCAGCAAAGTTCGATGCTA
TATTCAGCAAGTTTGGCAAAACTCGGCCAAATGCTTTGTCAGAAGATGAGATTAACGCCATGCTTAAACACAACCGC
AATATGTATGATTTTCTGGGCTGGGCCGCAGCAAACCTGGAATGGAAGTTGCTGCACAAAGTGGCAAAGGACAACGA
AGGCTTTTTGCAGCGAGAAATCGTGAGGGGCGCCTTCGATGGCAGCCTGTTCGAGCGTCTGCAGGAAAGCAAGAAAT
CTACCTGAATGTGGCAGTGAGCCGCGCGCAGTCGTCTATAAAACATCTGCACTTGAAATTTGAAAAGCCCTCCCTGC
ATTGTAGTTTTAGTTGTGTACGTCAAATAAGAAGTGTTTGGCCCGTCTACGGCGGCCTAAACATTTTCATTTGTATT
GTTAGTTTGAACTGGAATTTGTATTGTTATTGGTAGCACACAGAAGGTGTACAGTGGAACCTGTAATGTGCACTTGT
CT

>Clo10 [organism=Secale cereal] EF-hand calcium binding protein, caleosin mRNA, full CDS

ACCGCCGTAGATCATTTGCTTTCGATCTTGACTCTCGAGATCAAGCAGCAAAGTGTGGTGCGCGCGCACAGAGCGTT
ATGGCGGAGGAAGCTAAGGCGCCCGCCGACGCAATGTCGACGGTGGCGGAGGCGGCGCCGGTGACGGCCGAGCGGCC
GGTCCGGGCAGACCTGGAGACACACCTCCCGAAGCCCTACTTGGCTCGAGCTCTGGTCGTGCCCGACGTGTACCATC
CTGAAGGAGAGAGCGGGGAAGGGCACGAGCACGGGCAGAAGAGCGTGCTTCAGCAGCATGTGGCCTTCTTCGACCTG
GATGGCGACGGCATCGTCTATCCCTGGGAAACTTACCGAGGAATGCGGGCATTGGGCTTCAACGTGATCGTATCGTT
CATATTGGCAATTGTCTTTAATATTGGTTTCAGCTTCCCAACTCGACCTAGCTGGATACCATCTCCCTTGTTGCCCA
TACACATCAAGAACATCCACAGGGGTAAGCATGGTAGCGATTCCTCAACTTATGACACGGAGGGGAGGTTCATGCCG
GTAAATTTCGAGGGTATCTTTAGCAAGTACGCCCGCACGGCGCCTGAAAAGCTTAGCTTTGGCGACATCTGGAGGAT
GACCGAGGGCAACCGGCTGAACTTCGACTTCATTGGATGGATTGTGAGCAAGGGGGAGTGGATACTGCTGTACGTGC
TTGCCAAGGACGAGGAAGGGTTCCTCTCCAGAGAAGCTGTTCGCCGCTGCTTCGATGGTAGCCTGTTCGAGTTCATC
GCCCAGCAACGGAGGGAAGCCCACGACAAGCAGCACTAGAGTGCAACGTTGTCATCTTCGTGAGTAGGACTGTAGAC
CAGATGGATTACCTCATGTATGCCTATGAAATACTAAGAGAGAACGAGTACGAAGTTCATAGAAT

>Clo11 [organism=Secale cereal] EF-hand calcium binding protein, caleosin mRNA, full CDS

AAGCAGTGGTATCAACGCAGAGTACATGGGGGTCACCGCCGTCGATTATCGCTTTCGATC
GGGAGATTAAGCAGCAAAGTGTGGTGCGCACAGAGAGACAGCGCGGGTAATGGCGGAGGA
AGATAAGGCGCCCACCGACGCAACGTCGACGGTGGCGGAGGCGGCGCCGGTGACAGCCGA
GCGGCCGGTCCGGGCAGACCTGGAGACGCACCTTCCGAAGCCCTACTTGGCTCGAGCTCT
GGCCGTGCCCGATGTGTACCATCCTGAAGGGGCCGTGGAGGGGCACGAACACGGCCAGAA
GAGCGTGCTGCAGCAGCATGTGTCCTTCTTCGACCTGGACGGCGACGGCATCATCTATCC
CTGGGAGACTTACGGAGGAATGCGGTCGCTGGGTTTCAACGTGATCTTGTGTTTTATAGC
TGCAATTGCCATAAATATTGGTCTGAGTTTCCCAACTCTACCTAGCTGGATACCATCTCC
CTTGTTCCCCATACACATCAAGAACATCCACAGGGATAAGCATGGCAGCGACTCCTCGAC
CTACGACACGGAGGGAAGGTTTATGCCGGTAAATTTCGAGAGCATCTTCAGTAAGTACGC
CCGTACGGCGCCGGACAAGCTCAGCTTTGGCGACATCTGGAGGATGACTGAAGGCAACCG
GCTGCAGTACGACTTCTTTGGATGGTTGGTGAGCAAGGGAGAGTGGATACTGCTGTACGT
GCTTGCCAAGGACGAGGATGGCTTCGTCTCCAGAGAAGCTGTTCGCCGTTGCTTCGATGG
TAGCCTGTTCGAGTTCATCGCCCAGCAAAGGAGGGAAGCCCACGAGAAGCAGCAGTAGAG
TCAGACAGGACAGAGTAGAGTGCGACGCTGTCATCTCGGAGCGTCTTCGATCGTGAGTAT
TTCAAATGTGGACCAGATTGATTACCTCATCATGTGTGCCTAGGAAATAAGAGACGAACG
AATACCAAGTTCAGAGGGCTGAAAAGTTGTACGCTTTTATATAAAGACACAAGAAAAATA
TGTTGTACACGTATCAATTGTTGGAGTATATAGACATCTGTGTATATGATATACTATGAT
TTGTATCATCTCATGCCTTATCTCTGAAAGAG
