# Supplementary material for: Characterization of the caleosin gene family in the Triticeae
Source: BMC Genomics. 2014 Mar 27;15(1):239. doi: 10.1186/1471-2164-15-239 (PMC3986672; doi:10.1186/1471-2164-15-239)
Supplement: Supplementary file 17 — Additional file 17: Corrected caleosin sequences from Hordeum vulgare, and Brachypodium distachyon. These versions of the sequences are not available in GenBank. (PDF 218 KB) [file 12864_2013_7045_MOESM17_ESM.pdf]

Additional file 17

Caleosin sequences not available in GenBank

The following sequences from *Hordeum vulgare* and *Brachypodium distachyon* were used in the analysis of the caleosin gene family and are not available in public databases.

Hordeum vulgare Hv-*Clo6* was corrected by PG by addition of 1 nt and can only be corrected at GB by the original submitters.
Hv-*Clo8* was assembled from ESTs by PG and is in the process of being submitted to GB as a third party annotation.

Brachypodium - Bradi1g70400 was extended by PG on the 5’ end. The sequence is not available in GB.

```
>Hv-Clo6 Hordeum vulgare, EF-hand calcium binding protein, caleosin (Clo6)
mRNA  correction to gi|326515641 |dbj|AK375872.1| Frame shift corrected by
addition at nt 384. Verified by GB ESTs FD520388.1,BU980096.1,BU980308.1
GGCACGCACGCAGACCCGGCCTCGATCTGTGGAAAGAAGAGGATAGATCGGGAGAGAGAGAGAGGAGGAG
GAATCGCCTGACACGATGGGCGCCACCGGCCAACGTCGGCTGTCATCTCTGCCCGCGGCGGCGGCGGCCG
CGCCTCTCCTGCTTCTGCTCGCCGTGTCCTCCTGGAGCCAGGCGGCGGCGTCGGCGGCGGCGGCTCCGGC
CTGGACGACGGACCTGGAGAAGCACGTGGCGTTCTTCGACACCGACAACGACGGCATCGTCACCTTCTCC
GAGACCGAGCAAGGGCTTCGTGCCATCGGTCTTGGAGTTCTCGAGGCCACGGCCAGCGCCACCCTCATCA
ACGGAGTCATCGGACCCAAGACCAGACCCGAAAATGCTACGACATCGAGGTTTGACATCTACATAGCCAAC
ATCCACAAAGGGATGCACGGAAGCGACAGTGGCTCGTACGACGCCCAAGGAAGGTTTGTCCCGGCCAAGT
TCAACGGCATATTCACCAGGTTCGCCAAGGTCGAGCCGAACGCCCTGAACGAGGCCGAGCTGGAGGCCAT
GCGCACTGCCAACAGGAAGGAGGGTGACTTCAAAGGATGGGCGGCGTCCAAGGCGGAGTGGGGTATGCTG
TACAACCTCGCCAAGGACAAGGACGGCTTCCTTGAGAAGAACACCGCGCGCACCGTCTACGACGGCAGCC
TCTTCCCGAAGCTGGTGAAGAAGGCGAGCTCATCTGGAAATTAACCGAGCATGATATGTATTTGTATTTG
TTTTTCGATATGAAATTTGTACTCCGTATTATCCGGGTCTTGTTCGTTGAGCATGAGCTGTGTTGTCCCT
GTACAACTGAAATTGAAATCTGTTAATGAAAACATAATTTACTGCTCC

>HV-Clo8 Hordeum vulgare, EF-hand calcium binding protein, caleosin (Clo8)
mRNA G10 derived from from ESTs, 1047 nt, CDS 137-779
AAGCCACTACCGCTTTCCCTCTCACCGCGCCACCTTGGCATAACCACACCGAGATATCCA
GCTGATCTCATTCTCCCCGCGACGCCGTCCTCCCGCCCCTCGATCGCCGGCCGGCCGAAA
CCATAGATCCATCCATGGGCTCCAAACCTGCGGAGACGGCAGCAGCTGGGAGCCAGCAGC
AGCAGCAGGCGGAGGAGTCCTCCCTGGCGGACGTGTACAACCACGAGCTGACGCCGCTGC
AGAAGCACGCCGCCTTCTTCGACAGGAACAGGGACGGCATCATCTACCCCTCCGAGACCT
ACCAGGGGCTACGCGCCATCGGCTGCGGCGTCGTCGTTTCCGCCGCCGGCGCCGCCTTCA
TCAACGCCTTCCTTGCGCCCACGACGGTGCCGGCGAACGTGAAGCCTCCGGCTTTCAAGT
TCCCGATCTACGTGAAGACCATTAAGCAGGGCAAGCATGGGAGTGACACAGACGTGTACG
ACACCAACGGAAGGTTTGTTCCTGAAAAGTTTGAGGAGATATTCAAGAAGCATGCCCACA
CCAGGCCTGATGCCCTAACAAGCAAAGAGCTGGGAGAGATGCTTAAAGCAAACAGGGACC
CTAAAGATTTCAAAGGACGGGTGGGTGCCTTTGGAGAGTGGAGACTTCTCTATGCGTTGT
GCAAAGATAAGGATGGATTTCTTCACAAGGAGACTGTCAAGGCGGTCTATGATGGCAGCG
TGTTTGAGAAGTTGGAGCGAGAAAAAAAGGAAGCTAAGGAATTTGCCAAGAAGAAATGAT
GAAATGTCCTCCGATACCAGCTTATTTGTGGTTCTGTGAAAGTATTTGTAGATTGGGGCA
TTGTGCTTGTGAGTTCGGTTTCTCAAACTAATGTTGTGGTCGTTTGATTTCATTTGTTTC
TGGTTTCATGTTTCCTGGAATGAGACTCGGAGCCTCATTTTAAATGTATAGAAACTGCTT
```

GCAATTTGGGTAATATTGTTTCGCATTTGTTTTTACATTTTACCTTTTGTTGTTATTCAT
TGCTTATCCCATCCGATCCTCCATTTT

>Bradi1g70400_G6R .1 - 24 nt added to 5′ end to extend ORF relative to the annotation at http://brachypodium.org;
atgctgtcatcgcaggaggcgacg
ATGGCTGGTCGGCGACGATCCTCCATAGAGGCTCCTCTGCAGCTGCCACCGGTGGCTGCT
CTTCTGCTTCTATGGATCTTTAGCTGGAACTGCTTTGTTGCGATTGGCTGCGAGCCTGCA
TTTTCTAGTGCTGCCGCGACCACCATTCACGGTGTTCTTGGTCCTAAAACAAGCCCGGCT
GATGCATTGCCCGGCTTAGAAATACACGTTGAGAATATCCACAAAGCTATGCATGGAAGT
GGTACAGGCGCACTAGATGCTAAAGGAAGATTTGTTCCCCAAAAGTTCGAGGAAATATTC
ACTAGCTTTGCCAAGATTCGACAAGATGCCTTGACATTTCCAGAGATACAGGCGATGCTC
GTAGCACATCGGGATATACTAGACCCGGCATCATGGGCTCCACCCCAGGCAGAGTGGGGG
CTAATATACACGCTTGCAAGTGATTGGCTTGGGTTTCTTCACAAGGACAGCGTTAGAGGA
ATATACGATGGAAGCGTGTTTACCAAGTTGGAGGAAAAGTGGAACCCCTCTCAAAGTGAC
ATGCGATGA
